# Why enterprise resource planning initiatives do succeed in the long run: A case-based causal network

**DOI:** 10.1371/journal.pone.0260798

**Published:** 2021-12-16

**Authors:** Pierluigi Zerbino, Davide Aloini, Riccardo Dulmin, Valeria Mininno

**Affiliations:** Department of Energy, Systems, Territory and Construction Engineering, University of Pisa, Largo Lucio Lazzarino, Pisa, Italy; American University of Sharjah, UNITED ARAB EMIRATES

## Abstract

Despite remarkable academic efforts, why Enterprise Resource Planning (ERP) post-implementation success occurs still remains elusive. A reason for this shortage may be the insufficient addressing of an ERP-specific interior boundary condition, *i*.*e*., the multi-stakeholder perspective, in explaining this phenomenon. This issue may entail a gap between how ERP success is supposed to occur and how ERP success may actually occur, leading to theoretical inconsistency when investigating its causal roots. Through a case-based, inductive approach, this manuscript presents an ERP success causal network that embeds the overlooked boundary condition and offers a theoretical explanation of why the most relevant observed causal relationships may occur. The results provide a deeper understanding of the ERP success causal mechanisms and informative managerial suggestions to steer ERP initiatives towards long-haul success.

## 1 Introduction

Despite the COVID-19 pandemic, the global Enterprise Resource Planning (ERP) market is experiencing a prosperous trend in 2021 [1]. In 2020, SAP, the ERP market leader, has registered a greater increase in turnover than expected [2] by harnessing the Cloud technologies [3]. During the last five years, ERP systems have been building new momentum on the wave of topical streams, such as postmodern ERP [4], Big Data [5], Internet of Things [6], Process Mining [7].

Notwithstanding this, ERP failure rates are notoriously high [8], ranging from 40% to 90% according to different failure meanings [9,10]. The costs associated with a failure an ERP initiative may put the whole organisation at stake [11]. In 2016, Gartner [12] estimated that the lack of capabilities to fulfil future postmodern ERP strategies may give rise to a widespread failure on the ERP cloud initiatives. The contrast between growing investments and high failure rates characterises the ERP conundrum: in spite of a wealth of research regarding ERP success, the scientific literature is still lacking a thorough comprehension of why ERP initiatives succeed. This brainteaser may be rooted in two reasons.

First, the ERP scientific literature has spent remarkable efforts on figuring out *what* ERP success is, *i*.*e*., which constructs may define it, and *how* it may occur, *i*.*e*., which patterns may link the constructs to each other, but has mostly neglected *why* the proposed relationships may be observed [13]. The little focus on *why* a phenomenon is expected to occur points out the lack of causal explanations for such a phenomenon [14,15]. In investigating a phenomenon and its implications, exploring *what* and *how* has mainly descriptive purposes tied to an empirically driven perspective [16]. Delving into the *why* may enrich this valuable approach with theoretically driven explanations of the phenomenon. Conversely, the lack of *why* limits the understanding of a phenomenon [16] and the development of conceptually rigorous insights into it [17–19].

Second, in attempting to define and explain ERP success, the scientific literature has rarely considered its multi-stakeholder nature. This multi-stakeholder perspective may embody an interior boundary condition under which success occurs. Overlooking it may widen the gap between the theoretical models that define and explain the phenomenon under study–ERP success, in our case–and the empirical phenomenon itself [20]. Exploring the *why* of a phenomenon on the basis of theoretical models that might not adequately fit the reality may lead to theoretical inconsistency, hindering knowledge cumulativeness in the corresponding research stream [21].

To tackle these issues, this paper aims at answering the following research question:

"*Why does ERP success occur*?"

In doing so, we refer to success in the long run, which is conceived in the ERP post-implementation phase called "onward/upward" by [22]. This phase begins after overcoming the performance dip that typically occurs during the shakedown phase and is associated with the potential achievement of most ERP benefits [22]. It is mainly at this stage that it is possible to effectively assess whether the attained results have lived up to the expectations. Thus, success notions such as implementation and project success are outside the scope of this work because the success of an Information System (IS) is "*not intended to understand implementation or project success*", which may be better studied by change and project management theories [23, p. 504]. We used the ERP life cycle by [22] as a theoretical reference for the development of this paper.

To answer the research question, we developed a multiple case study based on the theory-building guidelines by [24]. From a managerial standpoint, to achieve our research objective may help decision makers in planning more accurate strategies for attaining the post-implementation benefits and in better addressing the use of resources during the ERP initiative.

The findings contribute to the ERP success scientific stream by showing that the continuous flow of ERP benefits may be achieved by the synergistic actions of three causes: the degree to which the ERP specifications are fulfilled over time; the user-system interaction, conceptualised as a cognition that diverges from frequency-oriented evaluations; and the continuous compliance between the attained benefits flow and the stakeholders’ expectations. This contrasts the predominant behavioural conceptualisation of system use and emphasises the role of the expectations and the longitudinal assessment of the system and information quality in attaining ERP success.

The remainder of this work is structured as follows: Section 2 provides a theoretical background; Section 3 expounds the research design; Section 4 illustrates the case findings; Section 5 discusses the results; Section 6 concludes the manuscript.

## 2 Theoretical background

This section reviews ERP post-implementation success to better highlight the research gap (2.1) and structures a theoretical framework for leading the development of this work (2.2).

### 2.1 ERP post-implementation success

ERP systems are commercial software packages that automate and integrate firm’s business processes [25]. Their implementation enables and often drives a business process reengineering based on best practices embedded in the software [26]. ERP systems are part of the Enterprise Systems [27], *i*.*e*., extensive organisation-wide applications including components such as ERP, Customer Relationship Management, Supply Chain Management, Product Lifecycle Management, Advanced Planning and Scheduling, Manufacturing Execution Systems, Business Intelligence and Data Analytics.

ERP systems are characterised by high integration–*i*.*e*., their parts are tightly amalgamated–and complexity–*i*.*e*., the relationships among their parts are numerous, nested, and interdependent [28]. Higher levels of integration and complexity usually correspond to a wider scope of the ERP initiative [28], which may thus gather interest and expectations from an increased number of stakeholders. This is a major reason why ERP success should be conceptualised from a multi-stakeholder standpoint [29–31], and not only from the perspective of the adopting firm and/or the end users. Example of additional stakeholders may be customers, suppliers, project leaders, key users, shareholders, vendors.

By drawing on [32], we define ERP post-implementation success as the effective exploitation of the ERP system and of the information it generates to achieve the intended benefits flow over time from the perspective of all the pertinent stakeholders. This excludes the technical installation success and all the related indicators (*e*.*g*., cost overruns, time estimates, project management metrics). Henceforth, for the sake of brevity, we use "ERP success" rather than "ERP post-implementation success". We make the following distinction regarding the benefits:

- Immediate benefits: benefits associated with the early effects of ERP-enabled business practices adoption and of modifications to the system and/or the information it generates. They directly affect profitability and are operational, *e*.*g*., reduction of labour, inventory, and quality costs.
- Future benefits: benefits associated with the mid- and long-term effective consolidation of the ERP-enabled business practices adoption and of modifications to the system and/or the information it generates. They include most of the benefits that indirectly affect profitability, such as managerial benefits (*e*.*g*., improved decision making), strategic benefits (*e*.*g*., support to growth plans), organisational benefits (*e*.*g*., facilitated organisational learning), and potential benefits (*e*.*g*., future ERP-enabled investment opportunities).

Table 1 reviews how the scientific literature has conceived and operationalised ERP post-implementation success. The papers were identified through a systematic review we conducted on Scopus using the following search string: ("ERP" OR "Enterprise Resource Planning" OR "Enterprise Systems" OR "Organisational-wide information system") AND (success OR benefits OR impacts). The query was restricted to journal papers. To grant comprehensiveness of the review, no restrictions on the year or type of publication were applied. Furthermore, the review was enriched by applying the snowballing method.

**Table 1:** Classification of the ERP post-implementation success scientific literature.

Perspective of analysis	Operationalisation	Reference	Theory of origin
Individual	User Satisfaction	[33–37]	--
	System Quality, Information Quality, Service Quality, Extended Use, User Satisfaction, Individual Benefits	[38]	IS success model by [39]
Organisational	Organisational benefits/impacts	[40–46]	--
		[47]	ES success model by [48]
		[25] (organisational benefits at plant level)	Organisational Information Processing Theory
		[49]	[25]
		[29,50–52]	ES benefits by [27]
		[53]	ERP benefits by [54]
	Information Quality, System Quality, Service Quality, Net Benefits	[30,55]	IS success model by [39]
	Information Quality, System Quality, Service Quality, Net Benefits, Financial Benefits	[56]	
	Achievement and ongoing improvement of the intended business results; Ease in adopting new ERP releases, other new Information Technologies, improved business practices, improved decision making	[57]	ERP experience cycle by [22]
	Organisational impact from a financial, customer, supplier, internal, and learning and growth perspectives	[58]	IS success model by [59]; balanced scorecard by [60]
	Vendor/Consultant Quality, Information Quality, System Quality, Individual Impact, Workgroup Impact, Organisational Impact	[32]	ES success model by [48]; IS success model by [59]
Organisational and Individual	Organisational benefits and User Satisfaction from the perspective of functional managers	[61]	IS success model by [59]
	User Satisfaction, Individual Impact, Organisational Impact, Intended Business Performance Improvement	[62]	
	System Quality, Information Quality, Service Quality, Intention to Use, User Satisfaction, Benefit of use, Business value	[63]	IS success model by [39]; User Information Satisfaction by [64]
	System Quality, Information Quality, Service Quality, Individual Impact, Workgroup Impact, Organisational Impact	[65]	IS success models by [39,59]; ES success model by [48]
	Vendor/Consultant Quality, Information Quality, System Quality, Individual Impact, Workgroup Impact, Organisational Impact	[66]	IS success model by [59]; ES success model by [48]
	Organisational Impact, User Satisfaction	[67]	IS success model by [48]
	System Quality, Service Quality, User Satisfaction, Individual Impacts, Net benefits	[68]	
	Information Quality, Individual Impact	[69]	
	Information Quality, System Quality, Service Quality, Individual Impact, Workgroup Impact, Organisational Impact	[70]	
	Information Quality, System Quality, Service Quality, Intention to Use / Use, User Satisfaction, Net Benefits	[71]	

The review we conducted highlights two evidences. First, surprisingly, only [53] probed *why* ERP success may occur. Despite the undoubted merit of their work, the ERP success definition they adopted coincides with ERP business benefits from the perspective of the implementing firm only. This overlooks the multi-stakeholder nature of ERP success. Moreover, their definition of post-implementation combines the shakedown and onward/upward phases. This may be debatable because, in these two phases, the conceptualisation of the success notion is deeply different [57].

Second, ERP success has been defined and described by drawing from the Information System (IS) success field. This was carried out in two ways. In the first one, the ERP literature used a single construct (*e*.*g*., User Satisfaction or Organisational Benefits) as a proxy for ERP success. This may be questionable because it is strongly subjective [72] and may lead to contrasting results [23]. In the second one, the ERP literature has borrowed and re-specified success models from the IS environment to the ERP one. Most papers equal ERP success to IS success explained through the IS success models by DeLone and McLean [39,59]. In particular, they generally leverage the quality of the information system, the quality of the information generated by the system, and the benefits or impacts yielded by the system to explain the success of the ERP. However, to the best of our knowledge, the ERP success literature has not adequately taken into account the multi-stakeholder perspective, despite its relevance to the topic. This finding may be worrying. Indeed, from a theory building perspective, the multi-stakeholder perspective is an interior boundary condition that may better specify the domain of a theoretical model, enhancing its adherence to the empirical system [20].

Therefore, the reason(s) why ERP post-implementation success occurs is still an open research question. A prominent reason for this may be that an ERP-specific interior boundary condition (*i*.*e*., the multi-stakeholder perspective) has often been discussed but not sufficiently considered in investigating the causal mechanisms conducive to ERP post-implementation success.

Thus, the next section sets a theoretical framework for investigating the reason(s) why ERP success may occur.

### 2.2 Theoretical framework

The awareness of commonalities and differences between IS failure and success has been effectively exploited for investigating the success of specific ISs, *e*.*g*., monitoring systems [73]. In particular, while recognising the causal asymmetry between IS success and failure, [73] set the simplifying hypothesis that "*the negated concept*, *ie*, *the lack of success*, *is the same thing as the opposite concept*, *ie*, *failure*" (p. 404). Indeed, to a certain extent, IS failure and success are linked to each other [74].

Although IS success and failure are two sides of the same coin, they are not totally specular because the relationships among variables describing IS phenomena do not necessarily assume causal symmetry [75]. IS failure may propagate in a domino effect [76], while success does not. "*With the benefit of hindsight one can usually reconstruct a systematic pattern of events that led to the failure*" [76, p. 284], while this is not true for success. Moreover, failure and success antecedents are not necessarily opposites [77]. Despite these differences, "*there can be multiple*, *equally effective pathways to IS success*" [73, p. 385], and this equifinality is valid for the IS failure too [76].

Lyytinen and Hirschheim [76] presented one of the most complete and spread empirical taxonomies of IS failure, consisting of four failure types:

- *Process failure*. It refers to two aspects of inadequate project management performance in developing an IS. First, the IS development process cannot create a workable system because of severe issues in the design, implementation, or configuration phases. Second, more frequently, the IS development process oversteps budget and/or time constraints.
- *Correspondence failure*. The IS design objectives expressed by the management are not met. It represents the management’s perspective of IS failure.
- *Interaction failure*. The users do not use the IS as intended or reject it because of their negative attitude towards it.
- *Expectation failure*. An IS may attract the attention of several stakeholders. This attention consists of expectations on how the IS will serve the stakeholders’ interest. Expectation failure occurs when the IS fails to meet a stakeholder group’s expectations. Thus, while Correspondence failure regards the system requirements expressed from the internal management perspective only, Expectation failure potentially voices the expectations of all the other stakeholders.

**Fig 1:**
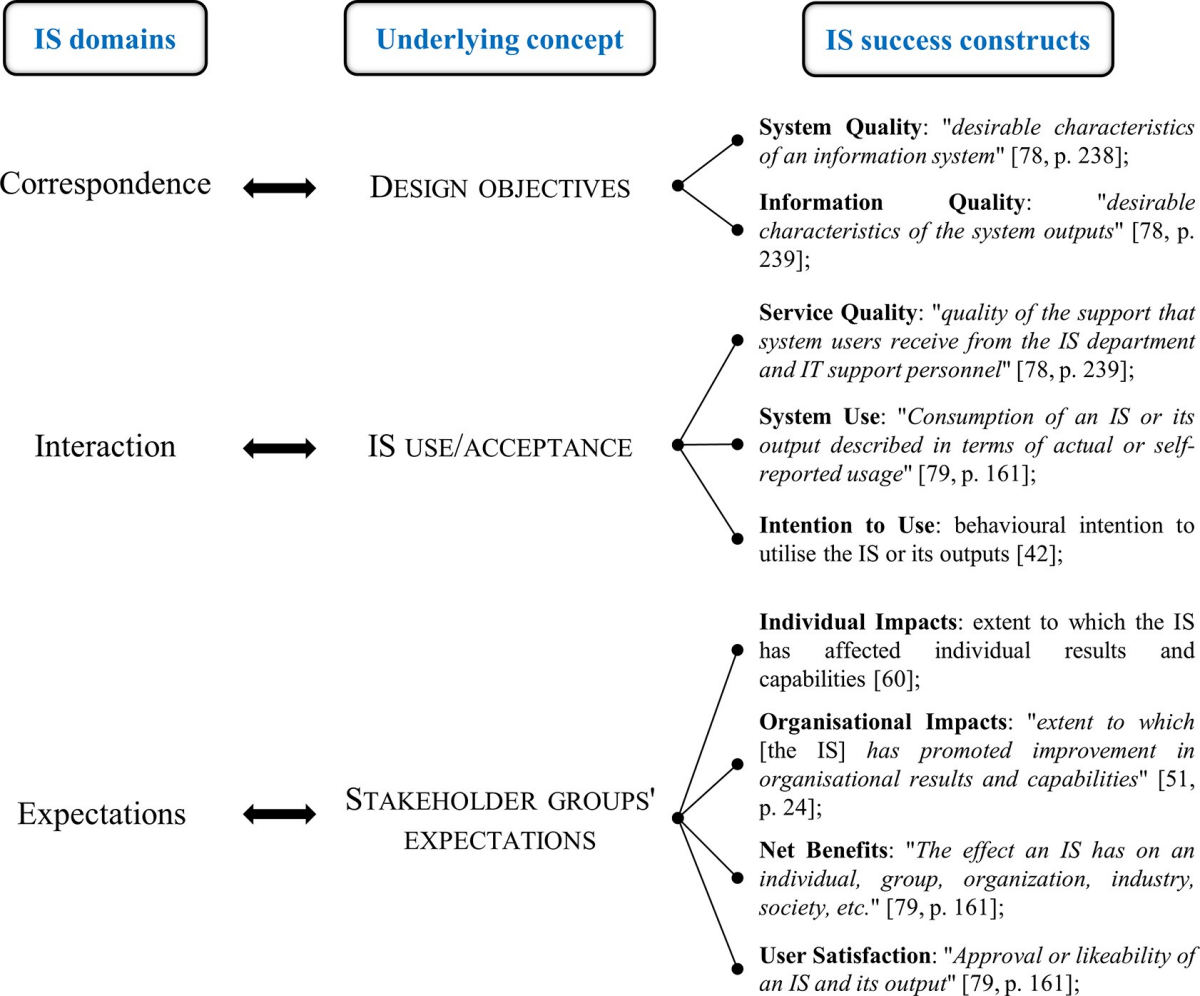
Association between IS domains and IS success constructs.

This IS failure taxonomy leverages four IS domains (Project, Correspondence, Interaction, Expectation) to classify several failures reported in the IS literature. The concepts underlying such domains are rather general: Project relates to time and/or budget; Correspondence to design objectives/specifications; Interaction to IS use/acceptance; Expectation to stakeholder group’s expectations. It is our contention that the four IS domain may be harnessed in a success context too. Indeed, the definition of the most prominent IS success constructs may be associated with the main concepts underlying such domains (Fig 1). We excluded the Process domain because if refers to a project management standpoint that is out of the scope of this manuscript. To identify the most prominent IS success constructs in Fig 1, we conducted a systematic literature on the IS success topic. Further detail on the protocol and the results of this review are provided in the Supporting Information file.

Hence, we argue that the Correspondence, Interaction, and Expectation domains may be framed as logical clusters according to which explain ERP success and collect the related evidences to answer our research question. A similar approach was adopted by [29], who used the four IS domains as a theoretical reference to define four ERP success categories and to integrate them into an ERP Critical Success Factors taxonomy. Thereby, the three selected domains are not intended to have any descriptive or explanatory power per se. Instead, their role is to offer a theoretical reference for steering the collection and arrangement of the evidences to pursue our inquiry.

## 3 Research design

To pursue our research objective, we adopted the case study methodology for theory building. Case research is always proper in IS research and appropriate at any stage of knowledge about a phenomenon, particularly when capturing the context of the phenomenon is of utmost importance [78]. We chose the inductive, positivist, case-based roadmap for theory building by [24] because of two reasons. First, the positivist epistemological approach aims at identifying the individual components of a phenomenon and at explaining them [78]. Second, inductive methods strongly relate to the development of explanations [79].

This section expounds the methodological choices underpinning the multiple case study. To facilitate its understandability, it was structured in three parts in line with the case research framework by [80, p. 172]: case selection (3.1), data collection (3.2), and data analysis (3.3).

### 3.1 Case selection

To define the case population, we considered only firms that completed the project phase. To exclude excessively small cases, we chose manufacturing firms with at least 250 system users and that challenged the same ERP implementation category [*cf*. 81]. We excluded the Vanilla implementations because of their small number of prospective users and limited scope and budget [81].

By following a geographical proximity criterion within the defined population, we selected four European cases, the unit of analysis of which was the whole ERP initiative. The number of cases provided sufficient analytical power [24]. To achieve both the theoretical replication and the literal replication, we investigated two cases in which the ERP initiative was in the advanced onward/upward phase and two cases in which it was at the very beginning of this phase. As regards the latter cases, our investigation proceeded until over two years after returning to "normal operations". Indeed, while success is mostly conceived within the advanced onward/upward phase, some operational concerns are strongly linked to the early period of this phase [30,31].

Company A is a worldwide Swedish–Swiss multinational corporation that operates in the field of automation and power. The plant in which we carried out the case study experienced an Oracle roll-out after being acquired by Company A.

Company B is an engineering and systems technologies international company that decided to replace its legacy system with a Microsoft Dynamics NAV ERP. Company B top managers were initially reluctant to adopt the ERP because wanted to retain the flexibility of their old system. Yet, they changed their mind because their existing ICT infrastructure was Microsoft-based.

Company C is an iron and steel international company that implemented an Infor ERP. After initial indecision, the top managers advocated the replacement of the old legacy system because it was not able to support their business anymore due to the lack of required functionalities.

Company D is an Italian national leader in manufacturing and installing cargo systems for liquified gas carriers. Its extant legacy system was not able to back up the firm’s growth and was replaced by a JD Edwards ERP solution.

Table 2 summarises the main characteristics of the ERP implementations.

**Table 2:** Characteristics of the selected firms.

Company Case	Sector	ERP Modules	Years after returning to "normal operation"
A	Electrical equipment	Finance; accounting; manufacturing; procurement; supply chain management; sales; production planning; quality management; inventory management;	Three years and eight months
B	Systems Engineering–both civil and defence applications	Finance; accounting; inventory management; quality management; project management; production orders; procurement and sourcing; sales;	Five years and half
C	Iron and steel	Finance; accounting; manufacturing; production planning; logistics; sales; inventory management;	Two years and two months
D	Iron and steel, cryogenic liquid storage tanks	Finance; accounting; quality management; inventory management; project management; sales and marketing;	Three years and half

### 3.2 Data collection

**Fig 2:**
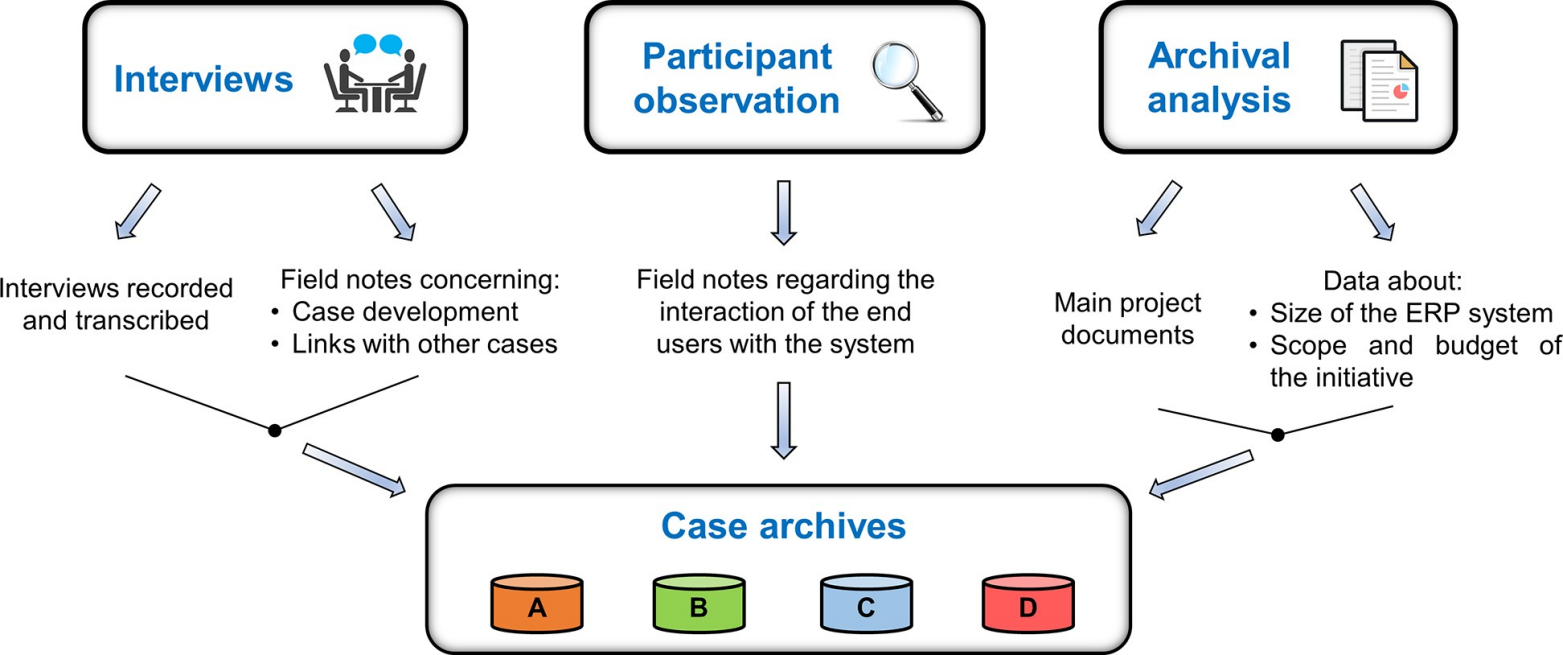
Data collection.

We developed a case study protocol according to [82]. Each case started with a kick-off meeting with the Project Sponsor, the Information and Communication Technology (ICT) Manager, and the Project Manager of the ERP initiative–if different from the ICT manager. During the kick-off meeting, commitment to the research project was guaranteed, visits were arranged, and the data collection methods were discussed and approved. Fig 2 summarises the data collection process, which started in April 2015 and ended in October 2020.

**Fig 3:**
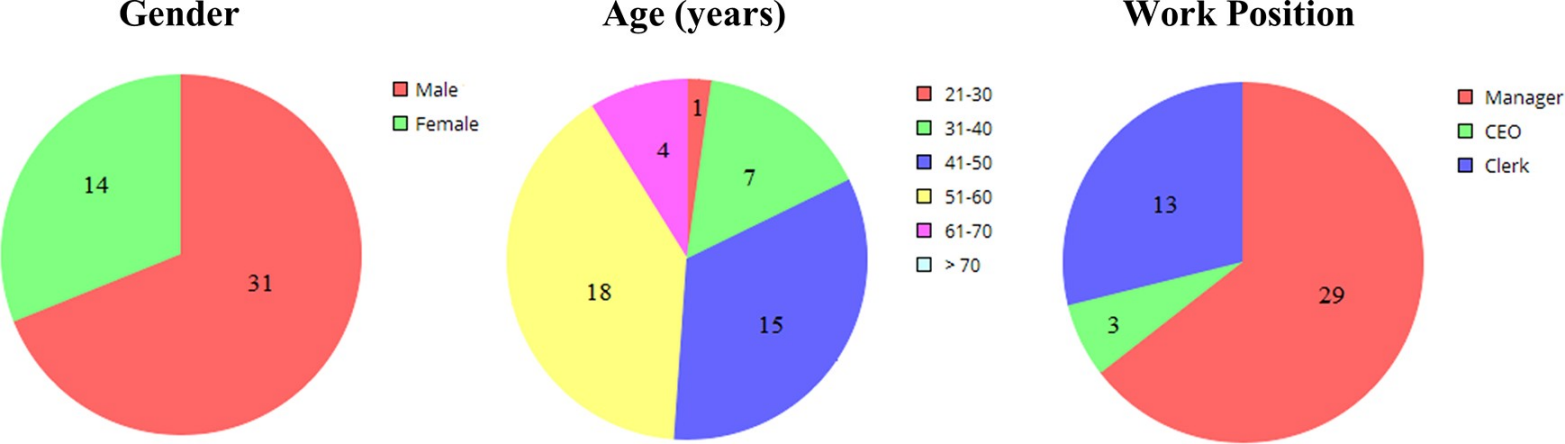
Details regarding the interviewees.

The data collection methods we relied on are:

- *Open-ended and semi-structured interviews* [79,83]. We conducted interviews concerning the overall perception of what ERP success may be and whom it may involve. To tackle the interview-related bias, we triangulated the data by multiple and heterogeneous data collection methods [82] and by conducting the inquiry from different functional perspectives [79]. The respondents were identified during the kick-off meeting based on both the implementation scope and the production approach of the firm. The authors were split into two teams. The interviews were conducted by the two teams alternately and were recorded and transcribed. One member of each team was the main interviewer and the other one recorded notes and observations. These roles were swapped after each interview. Table 3 presents an overview of the interviews and of the site visits, while Fig 3 details gender, age, and work position of the 45 interviewees. The average time length of the interviews was 1.5–3 hours. The question stems of the semi-structured interviews are in the Supporting Information file. Written informed consent was obtained for participation in the study beforehand. All the data involved in the study were anonymized and used only for the purposes of this study.

**Table 3:** Details on interviews and site visits.

Company Case	Number of site visits	Total number of interviews	Respondents
A	5	15	ICT, Project, Production Planning, Manufacturing, Finance and Accounting, Sourcing and SCM managers; One Quality Management Key User; one Logistics Key User; One shareholder (a local industrial logistics company); An external Key User from an engine partner supplier; One high-end customer;
B	4	15	ICT and Project, Sourcing, Make-to-Order Manufacturing, Engineer-to-Order Manufacturing, Accounting, Contract, and Finance managers; One Finance Key User; One external Key User from an electronic component partner supplier; One shareholder (private equity fund); Vendor-side Project Manager;
C	2	15	ICT and Project, Sales, Accounting, Manufacturing, Finance, and Logistics managers; One Manufacturing Key User, one Accounting Key User; One external Key User from a partner customer; One shareholder (a major pipeline manufacturer); Vendor-side Project Manager;
D	4	15	ICT, Project, Accounting, Manufacturing, Quality, Inventory, and Finance managers; One ICT Key User; One Accounting Key User; One external Key User from a partner customer (maritime logistics company); One shareholder (local bank); Vendor-side Project Manager;

Personal notes by the investigators regarding the on-going development of the case and the links with other cases were recorded in field notes [78].

- *Participant observation* [82,84]. We employed the participant observation method for observing the end users interacting with the ERP system in their daily routine, according to the guidelines by [85]. For each functional area in each case, the corresponding manager suggested two end users to observe, and the observation was performed until saturation.
- *Archival records* [82]. For each case, we analysed the main project documents. In addition, as suggested by [83], we probed quantitative data on the ERP initiatives, particularly about the size of the ERP system, scope, and budget.

### 3.3 Data analysis

**Fig 4:**
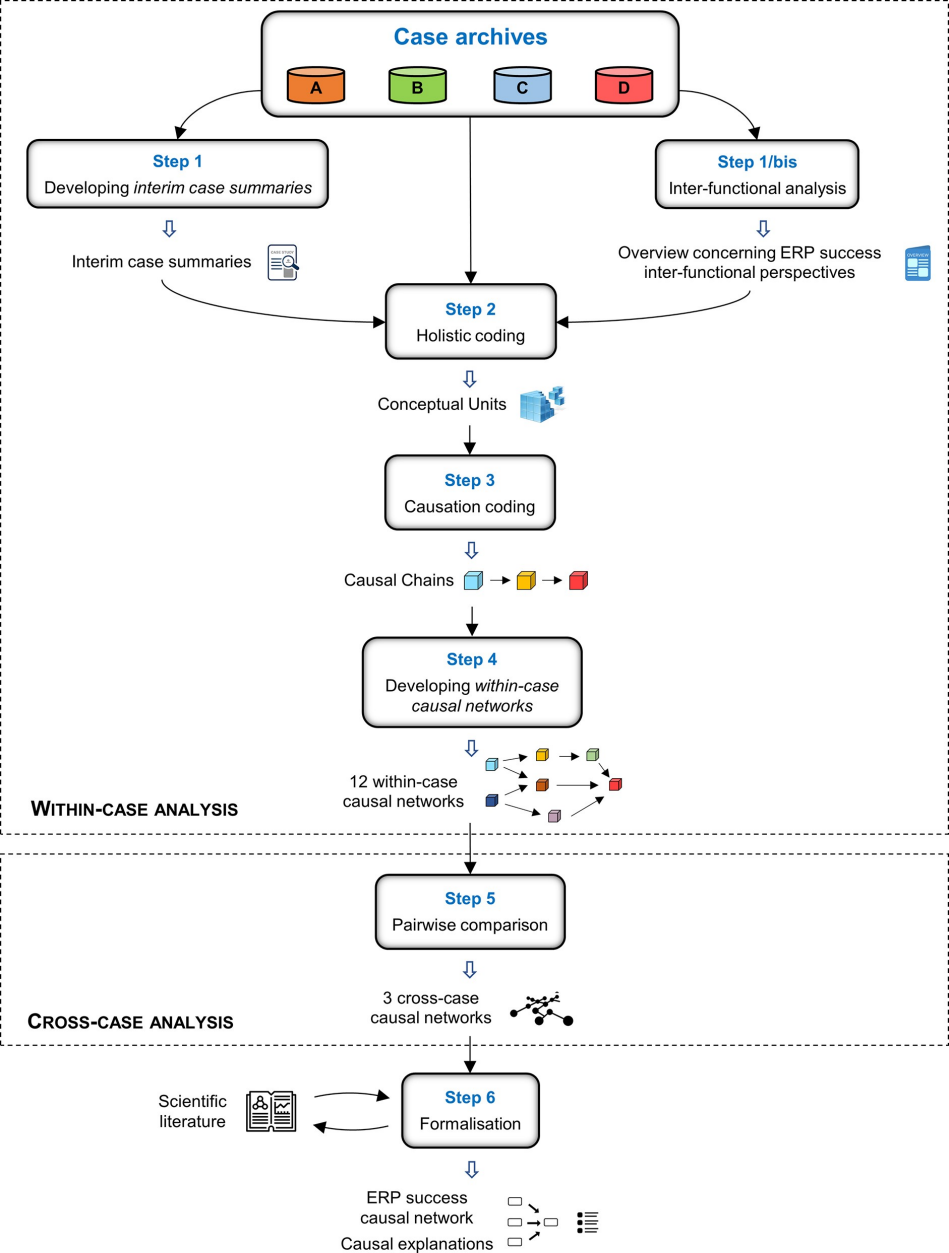
Data analysis flowchart.

The data analysis (Fig 4) was arranged in a two-stage way: within-case analysis (Steps 1–4) to develop a set of causal explanations for figuring out the mechanisms that led to ERP success; and cross-case analysis (Step 5) to cross the results from the within-case analyses for trying to generalise the findings.

The data analysis steps in Fig 4 are detailed in the following:

*Step 1*: *developing interim case summaries*. Drawing from the case archives, an interim case summary [86] was developed to synthesise and review the case data and findings.
*Step 1/bis*: *inter-functional analysis*. For each case, we compared the evidences stemmed from the different business functions by contrast tables [86] for obtaining an inter-functional overview concerning the success topic across the three logical clusters (*i*.*e*., Correspondence, Interaction, Expectation).
*Step 2*: *holistic coding*. The purpose of the holistic coding step [86] was to elicit the Conceptual Units from the case evidences. A Conceptual Unit is the main concept expressed by a unit of case data and appropriately labelled. Table A1 in Appendix A in the S1 File reports some examples of holistic coding. The outcomes from this step were jointly discussed among the authors to reach consensus regarding the sixty-one identified Conceptual Units.
*Step 3*: *causation coding*. We formalised the causal sequences, called Causal Chains (CCs), among the sixty-one Conceptual Units by causation coding [86]. A CC is a plausible, causal link between two or more Conceptual Units. Appendix B in the S1 File contains Table B1, which displays an example of the CCs we developed from case B, and Figure B1, which graphically illustrates how they may be used. Appendix B in S1 File also includes further details on how Conceptual Units form CCs.
*Step 4*: *developing within-case causal networks*. By combining all the CCs from the four cases and the three logical clusters, we developed twelve within-case causal networks. A within-case causal network is a graphical representation of multiple CCs and its purpose is to group and illustrate the causal relationships stemmed from the cases.
*Step 5*: *pairwise comparison*. The within-case causal networks were pairwise compared to assess their similarities and differences and to synthesise them into three cross-case causal networks (one for each logical cluster).
*Step 6*: *formalisation*. By combining the three cross-case causal networks, reported in the Supporting Information file, we developed the overall ERP success causal network. Furthermore, we discussed how the resulting network embedded the multi-stakeholder boundary condition, why the main causal relationships were observed, and the theoretical and managerial implications.

## 4. Results

This section briefly reports the results from the within-case analysis (4.1) and illustrates the results from the cross-case one (4.2).

### 4.1 Within-case analysis results

The four manufacturing firms are characterised by different approaches and challenged the ERP implementation for different reasons. Despite some operational issues, all of them overcame the shakedown phase and are currently benefiting from their new system. Table 4 describes the company cases.

**Table 4:** Description of the cases.

Case	Description
A	Company A is multinational corporation with US$ 30 billion revenue and over 100000 employees. It mainly operates in the electrical equipment field and also in the robotics, discrete automation, and power grid ones. In addition, it provides services ranging from engineering and consulting to installation, commissioning, maintenance, and training. Company A acquired the plant in which the case was developed and forced it to implement the ERP of the mother company to align the economic-financial reporting. Thus, the acquired site had to challenge a transition from an SAP ERP, which was working fine, to an Oracle one. The project manager that followed the SAP implementation was still working in the site and leveraged his previous experience to facilitate the new ERP initiative. Notwithstanding, he covered the role of Key User only because of his relatively little knowledge concerning Oracle. The new mother company appointed an external project manager, which led a team consisting of seven functional managers, one superuser (the finance and accounting manager), and some additional key users.
B	Company B is an engineering and systems technologies international company, with subsidiaries in four continents. It provides both products and engineering and consulting services in electromagnetic engineering, avionics, unmanned systems, and radar systems for both defence and civil solutions. To enhance its competitive capabilities, in 2010 the company has started a series of acquisitions and merging of affiliate or joint venture companies. During this process, it decided to replace its legacy system that was outdated, slow (*i*.*e*., high response times), and not able to measure some project performances, which were evaluated separately through Excel. One of the main requirements of the company was to select an ERP flexible enough to support the service provision and all its production approaches, *i*.*e*., make to order, make to stock, and engineer to order. After an intense scouting phase, the company chose Microsoft Dynamics NAV because it met the needed requirements and because the ICT infrastructure of the company was mostly based on Microsoft products. The project team consisted of eight functional managers and additional key users and was guided by the ICT manager, which was appointed as Project manager. The site in which we developed the case is the company headquarters.
C	Company C is an iron and steel international company with four factories in three continents. It produces pipes, coils, sheets, and extrusions, and has its own sales agent network to sell its products. To improve the accounting activities and the inventory management and to better align them with the production planning, it decided to implement an ERP by Infor. The site in which we conducted the case study is the headquarters and main factory of the company. The project team has been led by the ICT manager and consisted of five functional managers and a number of additional key users. Since this has been the first ERP implementation for the company, the project phase was tougher than expected because the resistance to change was very high. Moreover, further time was needed to reconfigure the hardware configuration because the high volumes of iron and steel within the main production facility reflected the wireless signal. During the end of the shakedown phase, the company was struggling with system response delays in using some sourcing functionalities.
D	Company D is a national leader in designing, constructing, supplying, and installing cargo systems for liquified gas carriers. The cargo systems are structured into three product lines differentiated according to the temperatures of the gas. In addition, the company produces heat exchangers, piping, and boilers. Since it mainly serves the petrochemical and the maritime sectors, it is equipped with several ballast tanks, carriers, and a ballasting barge whose maximum capacity exceeds 1000 tons. The company’s production approach is engineer to order. The decision to challenge an ERP implementation arose because the legacy system was not able to support the growth of the company and because its reporting and inventory management functionalities had become inadequate in light of the firm’s expansions. After a six-month scouting phase, the company chose the JD Edwards ERP solution for aiming at two objectives: to extend the digitalisation of the business processes; and to streamline and enhance the management of their design and manufacturing activities for yielding more accurate quotes. Company D experienced a phased implementation that favoured a smooth transition to the new system. The project team consisted of six functional managers, supported by some additional key users, and was led by the marketing manager, which has a strong background in project management. Since the feeling about the ERP in the shakedown phase was rather positive despite some operational issues, the top management is taking into consideration the implementation of the business analytics module.

The four ERP initiatives are judged as successful according the respondents. Although the point in time in which some benefits were perceived has been different than expected, the overall benefits flow has been satisfying.

For the sake of brevity, we did not include the within-case causal networks because they are an intermediate result that is included within the cross-case analysis. Despite the differences among the cases, no idiosyncratic Conceptual Units or CCs were found.

### 4.2 Cross-case analysis results

**Fig 5:**
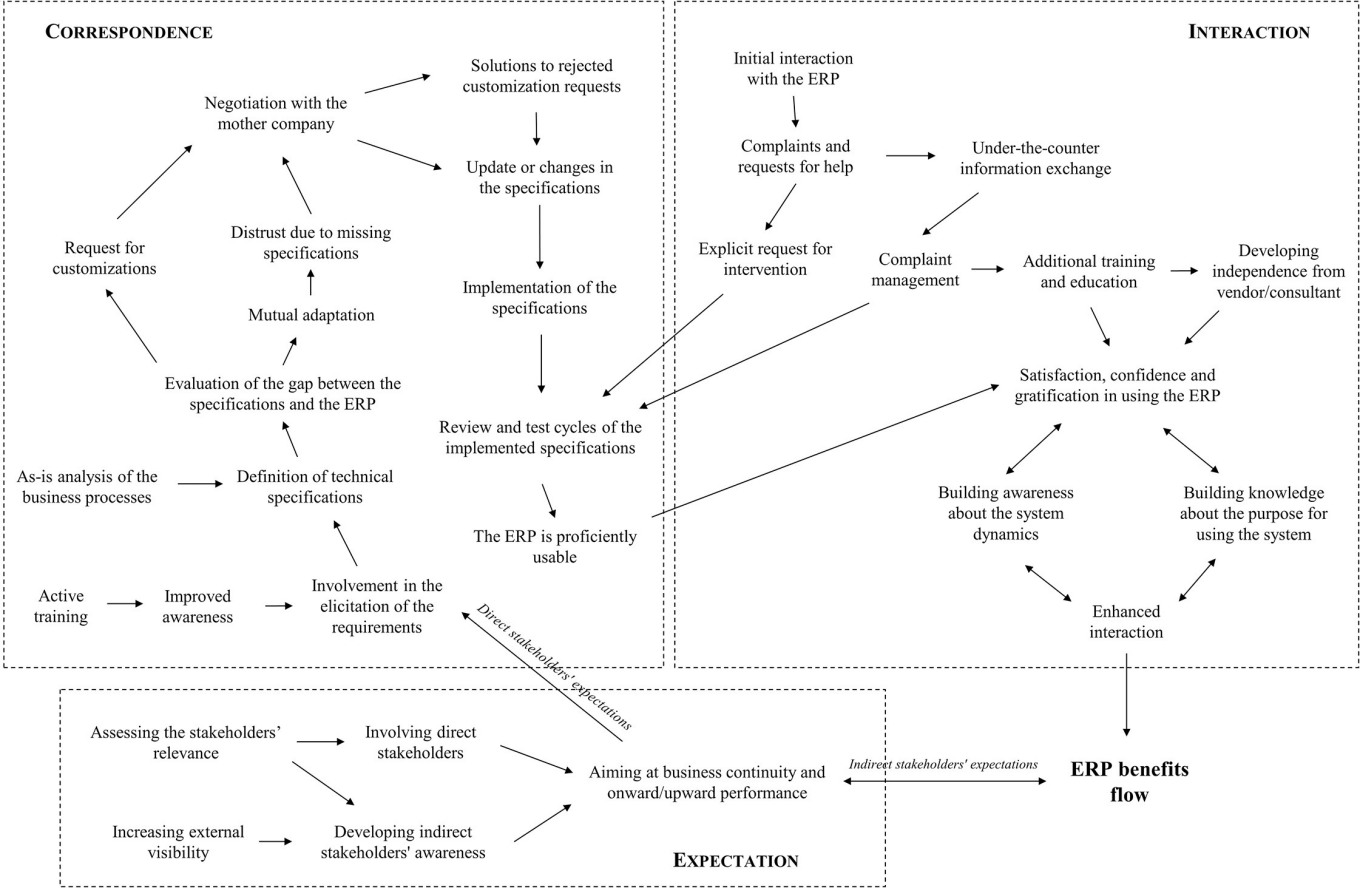
The ERP success causal network.

The overall ERP success causal network in Fig 5 contains a wealth of information on plausible causal mechanisms leading to ERP success. For the sake of brevity, this section focuses on the most relevant findings concerning the role of the Correspondence (4.2.1), Interaction (4.2.2), and Expectation (4.2.3) Conceptual Units in explaining ERP success.

#### 4.2.1 Correspondence cluster

The realisation of a proficiently usable ERP is strongly driven by the satisfactory implementation of the specifications, which regard the system, the needed input, and the desired output and which emerge from the as-is and to-be analysis of the business processes. Thus, the specification implementation, review, and test cycles are the core concepts in the Correspondence cluster.

The ERP specifications should not be considered as indisputable data over time because they may be reasonable only in the specific point in time which they were approved in, based on the information available in that moment. Some of them may be successfully tested and be still valid in the shakedown phase, as occurred in case D. They may be successfully tested, but changed later in the shakedown phase because of operational issues, as highlighted by a Key User from the manufacturing function in Case C:

"*We need to push the materials into production by the system as fast as possible because our production peak occurs in the summer* […]. *They* [the functional managers] *satisfied our requests*. *Two months later*, *during the summer*, [name retained for privacy] *started yelling with the keyboard in his hands because the system was still too slow*."

Moreover, some specifications may be successfully tested and still valid in the shakedown phase, but partly inadequate in the onward/upward phase–as stemmed from case A. The reasons for this latter occurrence are the unlikelihood to conduct the functional and non-functional tests in a complete way in all the possible scenarios and the difficulty in evaluating some specifications until after utilising the ERP massively.

Furthermore, some specifications may change over time during the onward/upward phase for responding to market needs, *e*.*g*., in case A, the company switched from standard management practices to lean production ones. Notably, the CIO from case B stated that:

"*The most important aspect is that the ERP includes the requested functionalities*. *The potential mistakes should not be linked to a wrong implementation but to a right implementation of specifications that*, *later*, *may turn out to be partly unsatisfactory and be modified/updated*".

More importantly, the fulfilment of the specifications over time is key to attain a benefits flow, as shown by some relevant case quotations in Table 5.

**Table 5:** Examples of case quotations on the requirements-benefits causal relationship over time.

Point in time	More benefits	Less benefits
Less than 8 months from the beginning of the post-implementation phase	"*It took no more than three months to ascertain that the purchasing order lead time was decreasing swiftly*. [The PM] *quarrelled for granting extra time for setting the requirements*. *We overdid*, *maybe*. *Some requests may have not been useful*, *but we managed to meet all of them*, *when possible*. *It was our best ERP implementation*, *hands down*" (Sourcing Manager, Case A). "*The new system was so… responsive*. *Its commands were different but not that hard*. […] *It was way easier to obtain the numbers I usually search for*. *To elaborate the second quarterly accounting report required at least 40% time less*." (Accounting Key User, Case D)	"*I asked for a different purchasing pattern for any order placed to our firm to speed up everything*. *Did you see it*? *No*? *Neither I did*. *The turning point was one year later*, *when they decided to meet our request and this shortened the purchasing and invoicing lead times*. *Why so later*?" (Partner supplier, Case B). "*I begged for two tree-based functionalities for facilitating our work*, *but they told me to be happy with only one of them*. *Our inventory management has become smoother and less complicated*, *but was it smart to renounce to something even better*?" (Inventory Manager, Case D)
More than 8 months after the beginning of the post-implementation phase	"*In 2017* [two years after the end of the shakedown phase], *the turnover of the company was experiencing a moderate growth*. *The new system already allowed to better manage a higher product differentiation*. *We decided to pour further capital in the firm to enter the drone market*. […] *They told me the system was the one to praise*. *I doubt it*. *I do not know how an electronic device may do this*. *Maybe it streamlined their way of working*, *but technology cannot do it alone*." (Shareholder, Case B) "*We were so pleased when they accepted* [to include a functionality to check the reserved stock]. […] *Now*, *we are jointly developing a module for ad-hoc maintenance of our production lines*, *with the possibility to collect sensor data from IoT*. […] *I was one of the main detractors of their choice to rely on a not-so-famous vendor*. *In the very end*, *I’m enthusiastic about having been wrong*.*"* (Partner Customer, Case C)	"*The new functionality* [to structure any engineer-to-order project by means of a modular approach] *helped us tremendously in classifying and arranging the developments by modules*. *During the first months since its implementation*, *it was rather glitched*. *We needed four months to have the issue fixed by the Indian guy*. *Once everything was fixed*, *we started to actually learn how to benefit from the ERP by not knowing which way to turn*, *by doing*, *by experience*." (Quality Manager, Case D) "[…] *we were mostly interested in their steady supply of batteries*. [An employee from firm A] *told us that the sourcing manager was delaying everything because he asked to modify some system customisations*. […] *We cannot halt our production because of a replaceable supplier*. *Somehow*, *after some difficulties*, *their supply became timelier*." (Customer, Case A)

Interestingly, the quotations in Table 5 show that the relationship between fulfilling the ERP requirements and achieving the benefits flow develops over time. During this time, the users may experience usage of the ERP and of its information, as suggested by the link between the Correspondence and the Interaction clusters in Fig 5. While this aspect is more evident in the following section, we argue that:

#### Finding 1

The system and information specifications may not be static over time even during the onward-upward phase and may causally affect the ERP benefits flow indirectly through the Interaction Conceptual Units.

#### 4.2.2 Interaction cluster

The user-system interaction may highlight the need to revise the implemented specifications to improve the usability of the ERP and of its information. This may occur during the shakedown phase, but also during the onward/upward phase after utilising the ERP for a while. In case A, the users of the logistics and finance functions experienced mediocre usability, despite appropriate vendor and package selection and the complete fulfilment of the specifications. The interaction was described as "intricate" to the extent that, after almost one year of the onward/upward phase, the Logistics Manager required additional human resources for managing the same activities:

"*After almost one year*, […] *four new employees were needed to perform our activities because this devilish system was muddled and not convincing*."

Interestingly, this opinion was quite the opposite during the test phases. Moreover, additional issues in the sourcing functionalities emerged later fortuitously, only when the sourcing users had to revise some sub-sourcing activities.

In case A, the usability was also negatively affected by the excessive number of unused functionalities. Most of them were specifically requested and/or customised, since they were considered as "*absolutely necessary to our daily routine*" by the key users and the process owners. Notwithstanding this, one year and half after the go-live, 30% of the overall functionalities were removed because they were not actually used, burdening the windows and the Graphical User Interface, entailing relevant albeit superfluous maintenance costs, and garbling the maintenance of other functionalities.

In line with this, several respondents from cases B and D, particularly those from the accounting and finance functions, underlined that an ERP system has its own life cycle and it is not advisable to squander it by poor usability and suboptimal configurations. In addition, the Chief Commercial Officer from case C stated that:

"*One may use an ERP that works as planned*, *but if s/he does so without smooth and serene utilisation*, *this may lead to abandon the system in the long run*".

ERP updates or modifications usually require users time to adapt by additional specific training and education activities. These activities make users more confident while using the ERP, and the more their use is acknowledged as correct, the more they build improved awareness and knowledge. In particular, the awareness concerns how the system works, how its information output is yielded, how to interpret the data, and which are the effects that user’s action exert on the business processes. The knowledge regards the link between the purpose (*e*.*g*., performing a task) for using the system and its information, on one hand, and the commands and functionalities for achieving this purpose, on the other hand.

These higher awareness and knowledge gratify the user and generate an enhanced interaction–an advanced form of interaction that is not meant as simply mechanical. In case B, the achievement of the advanced interaction required almost two years over the end of the shakedown phase. On one side, it entailed users realising the ERP-enabled improvements in their daily operations. On the other side, it allowed them to detect a mistake in the elaboration of an index for the production order management. In case A, under the same circumstances, the users discovered a function for solving misalignments between purchase orders and work orders. In case C, two users found a licit shortcut in the ERP for avoiding using a bugged functionality. Interestingly, the vendor-side Project Manager in case D stated that:

"*The interaction with the ERP must be appropriate*, *where appropriate means to use the system in a way to avoid not to use the system*. *It is necessary to understand how the data steer the execution of the processes* […] *or the processes may fail even if the ERP works fine*".

Fig 5 exhibits a causal effect of this enhanced interaction on the ERP benefits flow. As shown by some case quotations in Table 6, this effect is stronger when the above-mentioned awareness and knowledge are higher or, in other words, when the interaction is further enhanced.

**Table 6:** Examples of case quotations on the enhanced interaction-benefits causal relationship over time.

Effect	Case quotations
More benefits	"*It is not something you may acquire by training or by learning ’click here*, *then here*, *select this*, *fill that’*. *When you have this overall view*, *it’s… different*. *You detect errors easier*, *you find shortcuts*. *When the RC form was still glitched*, *I figured out how to bypass the glitch by increasing a parameter that usually depends on the results from the MRP*. *In the very end*, *everything was correct*." (Accounting Key User, Case C) "*When I see* [the system] *running*, *I think of how much data you have to feed it with to obtain the planning data*. […] *When you become aware of this while doing your work*, *results become different to your eyes*. *All the accuracy comes from there*." (Production Planning Manager, Case A) "*To use the system actually means a lot of things*. *You know how the puzzle of modules works*. *You know how the information flows along the ’modules pipeline’*. *You know what*, *when*, *why*, *how better than others*. *When this happens*, *it’s like having oil that greases all the ERP gears and this is felt at all the levels*. *Managers become happier*, *we become happier*, *users become more satisfied*, *the business goes forward smoother and better*." (Vendor, case D)
Less benefits	"*No use*, *no advantages*. *If you own a Ferrari and you don’t use it*, *you cannot truly appreciate it*. […] *During a post-implementation training camp*, *we found out that two workers were working pretty inefficiently because they were simply using the system in a mechanical way*. *We should have been nearer to them*. *We needed additional efforts to fix the issues their involuntarily caused*. *We lost time rather than saving it*." (Finance Manager, Case B) "[Company C] *itself was questioning the reliability of both our reserved stock level and of the estimated delivery date because they were unsure about how those numbers were popping out from their system*." (Partner Customer, Case C) "*After a while*, *I started refusing some informal help requests people were asking for*. *They were not actual requests for help*, *but a way to avoid figuring out something more about the ERP*. *This behaviour was slowing down the ’absorption’ of the new system* […]. *That’s why*, *maybe*, *improvements in formulating order cost estimates were lagging*." (Key User, Case D)

Accordingly, we contend that:

#### Finding 2

User-system interaction, when developed by building awareness about using the system and its information and knowledge concerning the purpose for using the ERP, may cause the achievement of ERP post-implementation benefits.

#### 4.2.3 Expectation cluster

The case evidences pointed out the usefulness of distinguishing between direct and indirect stakeholders. Direct stakeholders are those who directly interact with the ERP, *i*.*e*. the users. They may include end end/key users, process owners, members of the project team, but also external actors, *e*.*g*. partner suppliers that may be allowed to use some ERP functionalities for checking a stock level (cases A and B) or for loading a Bill of Materials (case D). For instance, an external Key User from a partner supplier in case A reported that:

"[Firm A] *procures our engines since years*. […] *I pushed for including a functionality in their system to grant us visibility of their engine stock levels*. *We did not want to disappoint each other*."

Another example of external actors is a partner customer that may exploit other functionalities to intervene in the product design (case B) or to check the reserved stocks (cases A and C).

Indirect stakeholders are actors who have expectations towards the ERP initiative, but that do not interact with the system. Indirect stakeholders should be informed of the status of the ERP initiative, or they would experience the consequences of the implementation without figuring out whether their expectations were not met. Table 7 contains some examples of indirect stakeholders suggested by the case informants.

**Table 7:** Examples of indirect stakeholders.

Case	Stakeholders
A	Company’s owners; shareholders; suppliers; system integrators; vendor; owners of processes not covered by the ERP scope;
B	Company’s owners; executive management; suppliers; customers (both internal and external); vendor, companies belonging to the same group and that may experience a rollout;
C	Shareholders; customers; suppliers; sales agents; owners of processes not covered by the ERP scope;
D	Company’s owners; shareholders; customers; suppliers; vendor; consultants.

Both direct and indirect stakeholders have expectations towards the ERP initiative, which are summarised by the *Aiming at business continuity and onward/upward performance* Conceptual Unit. They type of performance to improve depends on the expectations that each stakeholder sets for the ERP initiative. Nonetheless, such expectations exert different effects on the ERP benefits flow. Direct stakeholders’ expectations cause ERP benefits indirectly by the translation of these expectations into requirements to fulfil, which eventually lead to *Enhanced interaction* through the Correspondence and Interaction CCs (Fig 5). Instead, indirect stakeholders’ expectations showed a mutual causal relationship with the ERP benefits flow. This seems to be rather trivial, since having an expectation is not enough to achieve a benefit by itself. Although this unexpected finding is discussed in the next section, we can state that:

#### Finding 3

Direct stakeholders’ expectations may indirectly cause ERP benefits by the enhanced interaction between the users and a proficiently usable ERP if these stakeholders are actively involved in the elicitation of the requirements.

#### Finding 4

Indirect stakeholders’ expectations and the flow of ERP benefits may causally affect each other.

It is worth underlying that we assume that all the expectations are realistic. This is a basic assumption hypothesising that the change management activities conducted during the implementation project phase have been successful. Basic assumptions may not be perfectly true, but provide leverage in understanding the phenomenon under investigation. As underlined by Burton-Jones & Grange (2013, p. 634), making basic assumptions is typical in building theoretical concepts also in other fields, e.g.: in Economics, assuming that humans are rational.

## 5. Discussion

This section argues why ERP success may occur (5.1) and discusses the scientific and managerial implications (5.2).

### 5.1 Why ERP success may occur in the long run

The analysis of the causal network in Fig 5 revealed that ERP success may be mainly caused by three conjoint mechanisms: first, the causal action of direct stakeholders’ expectations on *Enhanced interaction* through *ERP proficient usability*; second, the causal effect of *Enhanced interaction* on *ERP benefits flow*; third, the mutual relationship between the indirect stakeholders’ expectations and *ERP benefits flow*.

As concerns the first causal mechanism, its explanation may be supported by the Expectation-Confirmation Theory (ECT). ECT posits that the match between the expectation towards a product or service prior to purchase and the performance perceived when using that product or service leads to post-purchase satisfaction, which in turn forms a repurchase intention [87]. Within the IS scope, ECT suggests that IS continuance is mainly explained by satisfaction with prior IS use, which stems from the consistency between the expectations over the IS and the perceptions after using the IS [88]. As pointed out by the ERP causal network, to satisfy direct stakeholders’ expectations means that the definition, review, and test cycles of the implemented specifications have fulfilled the established requirements. The initial interaction with the ERP developed in this way may entail further revising the specifications, which improves the proficient usability of the ERP. This increases the consistency between the expectations that direct stakeholders have towards the ERP and their judgement on the ERP. According to ECT, such a consistency makes users satisfied and more likely to continue using the ERP [*cf*. 89]. As showed by our findings, the prolonged interaction between a proficiently usable ERP and trained and satisfied users allows users to make cognitive progresses regarding ERP-related awareness and knowledge while performing a task. In doing so, users master the ERP and become further gratified and satisfied. This higher, aware, and knowledgeable satisfaction over time generates the enhanced interaction and users’ willingness to use the ERP at their best.

The second causal mechanism requires explaining why *Enhanced interaction* may cause *ERP benefits flow*. This mechanism may be explained by the Theory of Effective Use (TEU) by [90]. TEU posits that the IS use founded on the task-user-system interdependence is a necessary and sufficient condition for attaining performance improvements. Analogously, the *Enhanced interaction* Conceptual Unit is meant as an interaction involving interdependence among system and information characteristics, task characteristics, and user skills and capabilities. In particular, Fig 5 displays that it is generated by a set of CCs that consider the proficient usability of the ERP system and information, user capabilities in the interaction with the ERP, and the user-task link while using the ERP for one or more purposes. Furthermore, TEU claims that, given a task and a system, the use value may vary according to the user’s capabilities, leading to variations in achieving benefits. This is consistent with the findings reported in Table 6, which stress that a more aware and knowledgeable use causally leads to more benefits. Therefore, the CCs generating the enhanced interaction Conceptual Unit and the rationale underpinning Effective Use notion are consistent with each other. This analogy may thus explain the interaction-caused ERP benefits flow we observed.

The third causal mechanism accounts for the causal effect of the indirect stakeholders’ expectations on the ERP benefits flow. Admittedly, it is unlikely that having expectations over ERP benefits may be a necessary and sufficient condition to attain them. This direct action is attributable to the enhanced interaction. Instead, indirect stakeholders’ expectations strengthen the commitment and the economic support towards the ERP: although this does not cause any benefit, it grants continuity of the ERP initiative. Thus, the synergistic action of such expectations and of the enhanced interaction may cause the achievement of progressive ERP benefits distributed over time, *i*.*e*., the attainment of a distributed flow of ERP benefits.

In addition, attaining ERP benefits may cause a positive effect on future direct and indirect stakeholders’ expectations because it highlights that the outcomes from the ERP initiative are satisfactory. The stakeholders become more certain about the usefulness of the ERP and more inclined to use and support it over time. In this regard, ECT overlooks that consumer’s expectations towards a product or a service often changes following the consumption experience [88]. In our case, the achievement of an ERP benefit is advantageous for one or more stakeholders, which directly experience the positive impacts of the ERP implementation. Consequently, their realistic expectations of future benefits become more optimistic because their cognitive processes set a new positive reference to guide their decision processes. The new optimistic expectations shape their positive attitude towards the ERP initiative and, thus, their direct or indirect support towards it, which is needed to guarantee the potential flow of future benefits. Conversely, although lower expectations do not necessarily prevent the achievement of a benefit, they may reduce the stakeholders’ support and the interest towards the ERP initiative. If the attainment of one or more benefits does not reshape any low expectation positively, the system might be abandoned.

### 5.2 Implications for theory and practice

The proposed explanations for the causal mechanisms conducive to ERP success have some implications for the ERP success research stream. First, in evaluating the quality of an ERP system and of the information it generates, *e*.*g*., through the renowned System Quality and Information Quality constructs [91], it may advisable to consider the degree to which the desirable system and information specifications are fulfilled over time. This introduces a temporal dimension in the evaluation to reflect the evidence that ERP specifications are not a long-lasting constraint to be verified in the go-live date only. Instead, their assessment should account for the potential interventions on the ERP to cope with future, contingent upgrades/changes in the system or in its information during the ERP whole life cycle. It is not uncommon for turbulent market environments to call for changes in the business requirements during the onward/upward phase, which may imply adjustments in the ERP system and/or in the business processes over time [92,93]. Although the quality aspects have largely been considered in the ERP success literature [*e*.*g*., 30,32,55,56,63,65,94,95], to the best of our knowledge their multiple evaluations over time have never been linked to any ERP success causal mechanism. Staehr and colleagues [53] cunningly posited that additional IS projects may leverage off the ERP system to drive ERP benefits over time. Yet, they did not draw any relationship between ERP success and the longitudinal evaluation of the system and information quality. Instead, according to our findings, this overlooked aspect is critical to guarantee logical and temporal continuity between direct stakeholders’ expectations, proficient usability, and enhanced interaction over time.

Second, our findings suggest that the user-system interaction should be conceptualised as a cognition to capture the user cognitive progresses regarding aware and knowledgeable use. This is relevant because some ERP benefits are obtained only by massive use over long time [27], and their achievement is also affected by the user cognitive changes needed to adapt to the ERP modifications. The ERP success literature has mostly considered user-system interaction as a behavioural form of use, which cannot explain ERP success because frequency-related measurement of user-system interaction do not necessarily entail success [96]. For instance, [56] dropped any use-related variable in investigating the role of Information Technology governance in driving the ERP success because they were “*not seen as a fitting dimension of success unless system use is voluntary*, *which is not the case for ERP*” (p. 260). Our findings confirm this. In addition, they suggest that re-conceptualising the use of the system and of the information it generates as a cognition rather than a behaviour may be more effective in explaining performance improvements [97]. This provides an alternative view on the user-system interaction issue (*cf*. Table 1). Indeed, the behavioural conceptualisation is not able to grasp the above-mentioned cognitive aspects based on the system-task-user interdependence, which is instead fundamental to include the ERP integration and complexity notions while conceiving the use of an ERP [98].

Third, the degree to which the realistic expectations of direct and indirect stakeholders are fulfilled does matter to the achievement of the ERP benefits flow. Although [99] found that creating and maintaining realistic expectations of future benefits may positively affect the level of perceived benefits, the ERP literature has not addressed the role of the expectations in explaining the benefits flow from a multi-stakeholder perspective. Yet, we found that the expectations-benefits compliance may causally ensure the necessary endorsement, funding, and active participation and interaction to attain the distributed flow of benefits over time. Thus, to address the multi-stakeholder perspective in theorising and assessing ERP success, such a compliance should not be overlooked.

From a managerial standpoint, our findings provide useful hints. First, users should be enabled to work with higher self-cognition and with increased system and information awareness and knowledge to beget a benefits-generative enhanced interaction. To satisfy both direct and indirect stakeholders, the enhanced interaction should be consistent over time and should be constantly intertwined with high ERP proficient usability, which should be assessed in multiple points in time according to the system life cycle (*e*.*g*., at fixed time intervals and after system every update). In this regard, the top management may design an incentive system to foster enhanced interaction, *e*.*g*., a system that grants benefits to the users that exhibit increased system awareness in carrying out their activities. Second, it is advisable to assess the expectations-benefits compliance over time to figure out if the Correspondence-Interaction-Expectation loops maximise the ERP benefits flow. This knowledge of the ERP success causal mechanisms may help firms in better addressing the managerial efforts, reducing both the squandered resources during the ERP life cycle and the variability in achieving the ERP benefits. In turn, this may positively affect the propensity towards any rollout initiative.

## 6. Conclusions

The outstanding efforts spent by academics and practitioners over the last twenty years have transformed the ERP phenomenon in a renowned and mature reality. Nonetheless, the ERP acronym is still associated not only with long-lasting holistic benefits, but also with episodes of echoing failures, financial meltdown, and notorious lawsuits. Despite some notably contributions [*e*.*g*., 53,100], why ERP initiatives do succeed in the long run is largely unaddressed. One of the main reasons for this gap may be the lack of the context-specific "multi-stakeholder perspective" boundary condition in addressing ERP success. This widens the gap between any theoretical explanation of ERP success and the empirical occurrence of such a phenomenon, and may imply theoretical inconsistency. To fill this gap, we adopted the inductive case-based theory-building methodology by [24] and different qualitative data analysis techniques by [86] to explain why ERP success may occur. Thereby, we developed an ERP success causal network that embeds the multi-stakeholder overlooked boundary condition.

According to the findings, we justified the occurrence of the main Causal Chains by leveraging the Expectation-Confirmation Theory and the Theory of Effective Use. We argue that ERP post-implementation success may occur because of the conjoint and synergistic action of causal mechanisms related to ERP specifications, users’ cognitions, and stakeholders’ expectations. Thereby, this manuscript contributes to the understanding of the *why* theoretical criterion of the ERP success and proposes some implications regarding the ERP post-implementation success theorisation.

Nonetheless, this work is not free from limitations. Despite its appropriateness in establishing relationships among variables, case research methodology cannot always specify the direction of causation [78]. Moreover, although four cases provide sufficient explanatory power to attempt theory building [24], additional cases would have implied higher confidence in the completeness of the theory. Although no idiosyncratic evidences were found, more cases may have further reduced the gap between the ERP success causal network and the empirical phenomenon under study. This is true for the number of interviews too, which could be increased. In increasing the number of the cases, the data collection may be extended to non-European implementations to take into consideration possible ERP cultural misfits [101]. In fact, the geographical context which the cases were developed in might affect the managerial conduct [102,103].

The possible avenues for further research are rooted in such limitations. First, additional case studies may be conducted to strengthen the internal validity of the conclusions. Second, the explanation of the causal mechanisms we analysed may be formalised into a success model that may be operationalised and tested. This may open the way to the empirical application of a validated model. Third, text mining tools may be used to automate the analysis of the interview data and, thus, to account for additional data sources and larger data samples.

## Supporting information

S1 File (DOCX)
