# Supplementary material for: Why enterprise resource planning initiatives do succeed in the long run: A case-based causal network
Source: PLoS One. 2021 Dec 16;16(12):e0260798. doi: 10.1371/journal.pone.0260798 (PMC8675769; doi:10.1371/journal.pone.0260798)
Supplement: S1 File — (DOCX) [file pone.0260798.s001.docx]

**Appendix A**

Table A1. Examples of holistic coding

| **Conceptual Unit** | Clear planning and re-planning cycles | Mutual adaptability | Usability of the system | Involving critical stakeholders |
| --- | --- | --- | --- | --- |
| **Excerpts from the cases** | *"To set a go-live date is needed to plan backwards, although overrunning the go-live date is almost obvious. Yet, setting such a date is truly important because several human resources – they key users in particular – were temporarily dismissed from their duties for 50% of their work time, were partly substituted by other people, and were assigned to the ERP implementation. This is only part of the domino effect that an ERP initiative exerts and it would be almost impossible to cope with it without thinking about a go-live date that, however, is not carved in the stone. You know, multiple planning activities of the ERP project may link the desires and the boundaries of the business functions: I am not sure whether it may guarantee a successful outcome of the whole initiative, but every planning effort sets the stage for everything else."* | *"Some mismatches and functional flaws were resolved by specific system functionalities which we were not aware of, but that we learned by using the ERP. Often, during the shakedown phase, it is hard to figure out that some specifications are incorrect – because, for instance, some programs, links, or transaction volumes are still missing – and you may understand it only by using the ERP massively. For example, during the shakedown phase, the ERP system requires 30-60 seconds to enter an order. Yet, later in the onward/upward phase, the time needed to do so increased because of the execution of concurrent functionalities, the creation of additional reports, and so on. Such an experience taught us that it is correct to adapt our business processes to the ERP suite we chose but that, sometimes, also the system has to be modified. In our case, the ERP requires 21-23 barcode scans for each goods entry/exit and fourteen people to manage the process: to simplify this procedure, we modified the system."* | *"We have an ERP that meets the specifications, and this is a good point. But, even if I have an IT background, I saw some signs that it was not over there, few months after the end of the shakedown. The number of the help requests increased suddenly because the users were thinking to be sufficiently expert in using the system. Yet, unforeseen circumstances and contingent exceptions showed some limits of the ERP, particularly in the excessive awkwardness in remembering the commands and in using some sub-functionalities. Mind you: we pushed to receive high-level training, justifying the extra budget, but I felt that a number of users and the system were distancing from each other, and this was absolutely negative.* […] *Through the right adjustments to the system and to the people mindset, my colleagues and me agreed that the whole work regarding the ERP was a more objective success; it was not so until these interventions, because both meeting the specifications and the occurrence of the first clues of positive outcomes were deceiving us. Nonetheless, the usability of the system was not that good and we understood it only later."*  […] *By the right adjustments to the system and to the people mindset, my colleagues and me agreed that the whole work regarding the ERP was a more objective success; not until these interventions, because meeting the specifications and the occurrence of the first clues of positive outcomes were deceiving us, but the usability of the system was not that good and we understood it only later.* | *"In that year, one of our most important suppliers of internal combustion engines did not ship us any good for over one month – and I want to stress that we are one of its most important customers within the geographical area. This happened because, after changing the ERP system, they were able to process neither the orders nor the deliveries. To avoid any production standstill, we were thinking about searching for new suppliers, despite the long-term relationship with our partner. Yet, we preferred waiting because our order was not that compelling. The issue was finally solver:* [the supplier] *thanked us and admitted that involving us in the decision table concerning their ERP implementation would have avoided some problems in developing the order interface. There is no logical reason for excluding the most important stakeholders from such high-impact decisions.* |

# Appendix B

This Appendix contains additional details on the Causal Chains (CCs) we developed from the case data and on how they were exploited to create a causal network. The CCs were labelled as CCi,j, where i = case which the CC was detected in (*i.e.* A, B, C, D), while j = logical cluster which the CC referred to (*i.e.* Correspondence, Interaction, Expectation). Table B1 provides an example of the CCs from case B regarding the Interaction cluster.

Table B1. Causal Chains from case B – Interaction cluster

| **Conceptual Units of the Interaction cluster** | **Progressive code** | **Causal Chains (CC_B,Interaction_)** |
| --- | --- | --- |
| Initial interaction with the ERP | 1 | 1 > 2* |
| Complaints and requests for help | 2 | 2 > 3; 2 > 4 |
| Under-the-counter information exchange | 3 | 3 > 5 |
| Explicit request for intervention | 4 | 4 > 12 |
| Complaint management | 5 | 5 > 6; 5 > 12 |
| Additional training and education | 6 | 6 > 7; 6 > 8 |
| Developing independence from vendor/consultant | 7 | 7 > 8 |
| Satisfaction, confidence, and gratification in using the ERP | 8 | 8 > 9; 8 > 10 |
| Building awareness about the system dynamics | 9 | 9 > 8; 9 > 11 |
| Building knowledge about the purpose for using the system | 10 | 10 > 8; 10 > 11 |
| Enhanced interaction | 11 | 11 > 14 |
| Review and test cycles of the implemented specification** | 12 |  |
| The ERP is proficiently usable** | 13 | 13 > 8 |
| ERP benefits flow** | 14 |  |
| * The "*>*" symbol means "leads to" in a causal meaning. | | |
| ** Conceptual Units external to the Interaction cluster but linked to some Interaction Conceptual Units | | |

The Conceptual Units in the first column of Table B1 are causally linked to each other according to the information in the second and in the third column. For instance, by drawing from the case evidences, "*Interaction with the ERP*" (progressive code 1 in the second column) is causally linked to "*Complaints and requests for help*" (progressive code 2). The third column formalises this relationship by the following notation: 1 > 2 (see key to symbol at the bottom of Table B1). This Causal Chain is represented in Figure B1 (a).


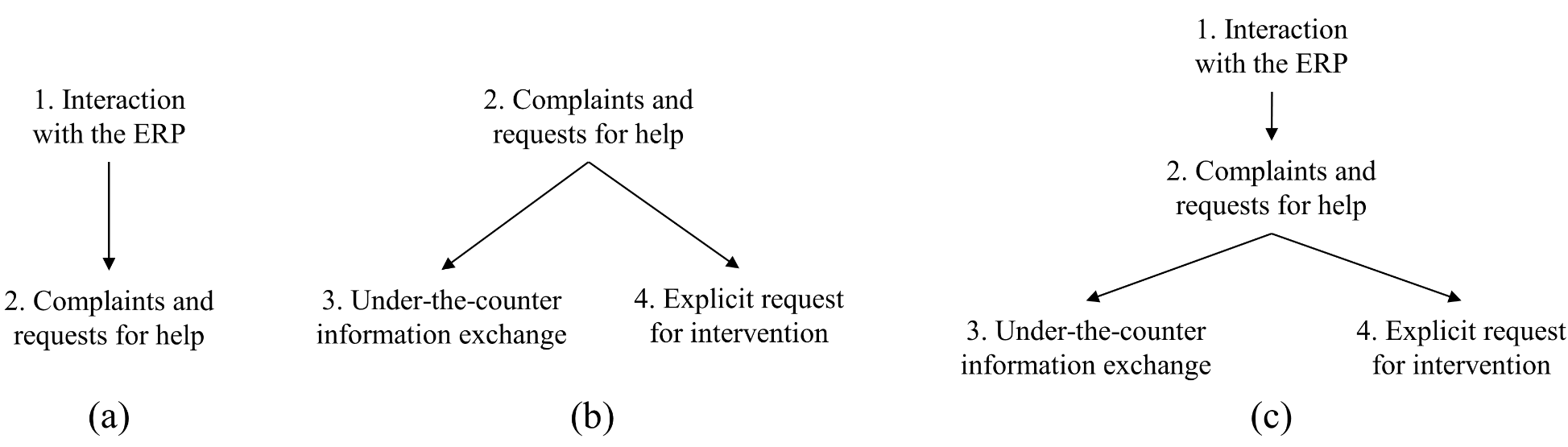


Figure B1. Progressive combination of the CCs from case B for the Interaction cluster

"*Complaints and requests for help*" is causally linked to "*Under-the-counter information exchange*" (progressive code 3) and to "*Explicit request for intervention*" (progressive code 4). These relationships are reported in column 3 of Table B1 as 2 > 3 and 2 > 4, respectively, and are shown in Figure B1 (b). Figure B1 (c) combines the CCs from Figure C1 (a) and (b) into a single chain. By following the sequence of the CCs in Table B1, it is possible to build the within-case causal network from case B for the Interaction cluster.

# Supplementary material

This section contains additional information that are too cumbersome to be included in the Manuscript file but that may be useful for the sake of completeness. In detail, it consists of three sub-sections. The first one (Literature Review on IS success) reports the literature review on the IS success topic we conducted to support the development of the ERP success theoretical framework in Section 2.2 of the Manuscript file. The second one (Cross-case causal networks) illustrates the cross-case causal networks of the Process, Correspondence, Interaction, and Expectation clusters. The third one (Questions of the semi-structured interviews) contains the questions posed during the semi-structured interviews.

## Literature Review on IS success

This section contains additional details pertaining to a systematic literature review that we developed to support the comparison between IS failure and IS success (see Section 2.2 in the Manuscript file). The objective of this review was to obtain an overview of the topic to grasp its main characteristics and to identify the most prominent IS success constructs.

We searched for "Information System Success" in "Title, Abstract, Keywords" on Scopus, limiting the query to journals only and to the following disciplines: Computer Science; Business, Management and Accounting; Social Sciences; Decision Sciences, Engineering. The timeframe was 1992-2020 because the most influential IS success model, *i.e.* DeLone & McLean (1992), was published in 1992 and it summarises the IS success literature prior to 1992. The number of resulting hits was 491. We focused only on those papers that needed to operationalise IS success, restricting the number of works to 147.

We found that: first, almost all the most notable developments in the IS success conceptualisation (*e.g.* Gable et al., 2008; Seddon, 1997) are a re-specification of the IS success models by DeLone & McLean (1992, 2003); second, out of 147 contributions that needed to operationalise IS success, 139 relied on one or more IS success constructs from the IS success models by DeLone and McLean. Thus, we can confidently state that the IS success constructs in Figure 1 of the Manuscript file are the most acknowledged and that the IS success literature is almost unanimous in considering the IS success models by DeLone and McLean as the most prominent and still current success models.

Table S1 reports the 139 papers that exploited one of more constructs from DeLone & McLean (1992, 2003) to operationalise IS success. The references of these papers are at the bottom of this file.

Table S1. Operationalisation of IS success

| IS success constructs | References |
| --- | --- |
| *System Quality* | Rainer Jr. and Watson (1995); Saarinen (1996); Li (1997); Seddon (1997); Drury and Farhoomand (1998); Kim et al. (1999a, b); Skok et al. (2001); Rai et al. (2002); McGill et al. (2003); DeLone and McLean (2004); Landrum and Prybutok (2004); Liu et al. (2005); Almutairi and Subramanina (2005); Iivari (2005); Bradley et al. (2006); Crowston et al. (2006); Wu and Wang (2006); Byrd et al. (2006); Lin and Hee (2006); Sabherwal et al. (2006); Kulkarni et al. (2006); Jang et al. (2006); Hussein et al. (2007); Lin (2007); Gable et al. (2008); Landrum et al. (2008); Petter et al. (2008); Hwang and Xu (2008); Yen et al. (2008); McGill and Klobas (2008); Wang (2008); Wang and Liao (2008); Lee et al. (2009); Chen and Cheng (2009); Rezaei et al. (2009a, b); Ong et al. (2009); Chung and Kwon (2009); Floroupolos et al. (2010); Almarashdeh et al. (2010); Landrum et al. (2010); Klobas and McGill (2010); Adeyinka and Mutula (2010); Jang (2010); Lee et al. (2011); Zhou (2011); Park et al. (2011); Lin and Wang (2012); Ayyash et al. (2012); Chen and Cao (2012); Yeh and Tao (2012); Khayun et al. (2012); Leung et al. (2012); Sørum et al. (2012); Kim et al. (2012); Eom et al. (2012); Al-Debei et al. (2013); Balaban et al. (2013); Keramati and Salehi (2013); Hollmann et al. (2013); Petter et al. (2013); Lin et al. (2013); Choi et al. (2013); Lwoga (2013); Poelsman et al. (2013); Lai (2014); Wang et al. (2014); Popovič et al. (2014); Mtebe and Raisamo (2014); Wang and Lu (2014); Ghobakhloo and Tang (2015); Cho et al. (2015); Xinli (2015); Rouibah et al. (2015); Huang et al. (2015); Chung et al. (2015); Hew et al. (2016); Sappri et al. (2016); Son et al. (2016); Chiu et al. (2016); Jagannathan et al. (2016); Wang et al. (2016); Ojo (2017); Gaardboe et al. (2017); Assegaff et al. (2017); van Cauter et al. (2017); Wie and Widjaja (2017); Shagari et al. (2017); Ramírez-Correa et al. (2017); Abrego Almazán et al. (2017); Lin et al. (2017); Laumer et al. (2017); Suh et al. (2017); Duan et al. (2017); Ji-fan Ren et al. (2017); Nugroho and Prasetyo (2018); Yakubu and Dasuki (2018); Daghouri et al. (2018); Aminah et al. (2018); Arenas-Gaitán et al. (2018); Aldholay et al. (2018a, b); Chatterjee et al. (2018); Widjaja et al. (2018); Efiloğlu Kurt (2019); Aldholay et al. (2019); Martins et al. (2019); Wang et al. (2019); Alzahrani et al. (2019); Al-Mamary (2019); Doleck et al. (2019); Harr et al. (2019); Tam et al. (2019); Barreiras et al. (2019); Motiwalla et al. (2019); Alkraiji (2020); Al-Fraihat et al. (2020); Lwoga et al. (2020); Bradford et al. (2020); Khayer et al. (2020); Yoo (2020); Chang et al. (2020); |
| *Information Quality* | Rainer Jr. and Watson (1995); Li (1997); Seddon (1997); Martinson and Chong (1999); Kim et al. (1999a, b); Skok et al. (2001); Ishman et al. (2001); Rai et al. (2002); McGill et al. (2003); DeLone and McLean (2004); Landrum and Prybutok (2004); Liu et al. (2005); Almutairi and Subramanina (2005); Iivari (2005); Bradley et al. (2006); Crowston et al. (2006); Wu and Wang (2006); Byrd et al. (2006); Lin and Hee (2006); Kulkarni et al. (2006); Jang et al. (2006); Hussein et al. (2007); Lin (2007); Gable et al. (2008); Landrum et al. (2008); Petter et al. (2008); Hwang and Xu (2008); Yen et al. (2008); Wang (2008); Wang and Liao (2008); Al-Adaileh (2008); Chen and Cheng (2009); Rezaei et al. (2009a, b); Ong et al. (2009); Chung and Kwon (2009); Floroupolos et al. (2010); Almarashdeh et al. (2010); Landrum et al. (2010); Klobas and McGill (2010); Fleischman et al. (2010); Adeyinka and Mutula (2010); Jang (2010); Lee et al. (2011); Zhou (2011); Park et al. (2011); Lin and Wang (2012); Ayyash et al. (2012); Chen and Cao (2012); Yeh and Tao (2012); Khayun et al. (2012); Leung et al. (2012); Sørum et al. (2012); Kim et al. (2012); Eom et al. (2012); Al-Debei et al. (2013); Balaban et al. (2013); Keramati and Salehi (2013); Hollmann et al. (2013); Petter et al. (2013); Lin et al. (2013); Choi et al. (2013); Lwoga (2013); Poelsman et al. (2013); Hazen et al. (2014); Lai (2014); Wang et al. (2014); Popovič et al. (2014); Mtebe and Raisamo (2014); Wang and Lu (2014); Ghobakhloo and Tang (2015); Cho et al. (2015); Xinli (2015); Rouibah et al. (2015); Huang et al. (2015); Chung et al. (2015); Hew et al. (2016); Sappri et al. (2016); Son et al. (2016); Chiu et al. (2016); Jagannathan et al. (2016); Wang et al. (2016); Ojo (2017); Gaardboe et al. (2017); Assegaff et al. (2017); van Cauter et al. (2017); Wie and Widjaja (2017); Shagari et al. (2017); Ramírez-Correa et al. (2017); Abrego Almazán et al. (2017); Lin et al. (2017); Laumer et al. (2017); Suh et al. (2017); Duan et al. (2017); Ji-fan Ren et al. (2017); Nugroho and Prasetyo (2018); Yakubu and Dasuki (2018); Daghouri et al. (2018); Arenas-Gaitán et al. (2018; Goeke et al. (2018); Aldholay et al. (2018a, b); Chatterjee et al. (2018); Widjaja et al. (2018); Efiloğlu Kurt (2019); Aldholay et al. (2019); Martins et al. (2019); Wang et al. (2019); Alzahrani et al. (2019); Al-Mamary (2019); Doleck et al. (2019); Harr et al. (2019); Tam et al. (2019; Barreiras et al. (2019); Motiwalla et al. (2019); Alkraiji (2020); Al-Fraihat et al. (2020); Lwoga et al. (2020); Bradford et al. (2020); Khayer et al. (2020); Yoo (2020); Chang et al. (2020); |
| *Service Quality* | Li (1997); Skok et al. (2001); DeLone and McLean (2004); Landrum and Prybutok (2004); Liu et al. (2005); Almutairi and Subramanina (2005); Lin and Hee (2006); Jang et al. (2006); Lin (2007); Landrum et al. (2008); Petter et al. (2008); Wang (2008); Wang and Liao (2008); Lee et al. (2009); Chen and Cheng (2009); Floroupolos et al. (2010); Almarashdeh et al. (2010); Landrum et al. (2010); Klobas and McGill (2010); Adeyinka and Mutula (2010); Jang (2010); Lee et al. (2011); Zhou (2011); Ayyash et al. (2012); Tella (2012); Yeh and Tao (2012); Khayun et al. (2012); Sørum et al. (2012); Al-Debei et al. (2013); Balaban et al. (2013); Keramati and Salehi (2013); Hollmann et al. (2013); Petter et al. (2013); Lin et al. (2013); Choi et al. (2013); Lwoga (2013); Poelsman et al. (2013); Gorla and Somers (2014); Wang et al. (2014); Mtebe and Raisamo (2014); Ghobakhloo and Tang (2015); Cho et al. (2015); Rouibah et al. (2015); Huang et al. (2015); Chung et al. (2015); Hew et al. (2016); Sappri et al. (2016); Sebetci and Çetin (2016); Son et al. (2016); Chiu et al. (2016); Wang et al. (2016); Ojo (2017); Assegaff et al. (2017); van Cauter et al. (2017); Wie and Widjaja (2017); Shagari et al. (2017); Abrego Almazán et al. (2017); Lin et al. (2017); Laumer et al. (2017); Suh et al. (2017); Nugroho and Prasetyo (2018); Yakubu and Dasuki (2018); Daghouri et al. (2018); Aminah et al. (2018); Aldholay et al. (2018a, b); Chatterjee et al. (2018); Widjaja et al. (2018); Efiloğlu Kurt (2019); Aldholay et al. (2019); Martins et al. (2019); Alzahrani et al. (2019); Al-Mamary (2019); Doleck et al. (2019); Harr et al. (2019); Tam et al. (2019); Motiwalla et al. (2019); Al-Fraihat et al. (2020); Lwoga et al. (2020); Bradford et al. (2020); Khayer et al. (2020); Yoo (2020); Chang et al. (2020); |
| *Use / Intention to use* | Szajna (1993); Rainer Jr. and Watson (1995); Saarinen (1996); Gelderman (1997); Martinson and Chong (1999); Skok et al. (2001); Rai et al. (2002); McGill et al. (2003); DeLone and McLean (2004); Liu et al. (2005); Almutairi and Subramanina (2005); Iivari (2005); Bradley et al. (2006); Wu and Wang (2006); Lin and Hee (2006); Sabherwal et al. (2006); Jang et al. (2006); Lin (2007); Landrum et al. (2008); Petter et al. (2008); Wang (2008); Wang and Liao (2008); Lee et al. (2009); Chen and Cheng (2009); Almarashdeh et al. (2010); Klobas and McGill (2010); Fleischman et al. (2010); Adeyinka and Mutula (2010); Jang (2010); Lee et al. (2011); Tella (2012); Chen and Cao (2012); Khayun et al. (2012); Leung et al. (2012); Eom et al. (2012); Al-Debei et al. (2013); Balaban et al. (2013); Keramati and Salehi (2013); Hollmann et al. (2013); Petter et al. (2013); Hazen et al. (2014); Wang et al. (2014); Mtebe and Raisamo (2014); Ghobakhloo and Tang (2015); Cho et al. (2015); Xinli (2015); Rouibah et al. (2015); Huang et al. (2015); Chung et al. (2015); Hew et al. (2016); Sebetci and Çetin (2016); Son et al. (2016); Borena and Negash (2016); Chiu et al. (2016); Wang et al. (2016); Ojo (2017); Gaardboe et al. (2017); Assegaff et al. (2017); van Cauter et al. (2017); Ramírez-Correa et al. (2017); Abrego Almazán et al. (2017); Lin et al. (2017); Suh et al. (2017); Yakubu and Dasuki (2018); Daghouri et al. (2018); Arenas-Gaitán et al. (2018; Goeke et al. (2018); Aldholay et al. (2018a, b); Chatterjee et al. (2018); Widjaja et al. (2018); Efiloğlu Kurt (2019); Aldholay et al. (2019); Martins et al. (2019); Wang et al. (2019); Alzahrani et al. (2019); Al-Mamary (2019); Doleck et al. (2019); Harr et al. (2019); Tam et al. (2019); Al-Fraihat et al. (2020); Lwoga et al. (2020); Bradford et al. (2020); Chang et al. (2020); |
| *Individual Impacts / Organisational impacts / Net Benefits* | Rainer Jr. and Watson (1995); Saarinen (1996); Li (1997); Seddon (1997); Drury and Farhoomand (1998); Martinson and Chong (1999); Kim et al. (1999a, b); Skok et al. (2001); Rai et al. (2002); McGill et al. (2003); DeLone and McLean (2004); Liu et al. (2005); Almutairi and Subramanina (2005); Iivari (2005); Crowston et al. (2006); Wu and Wang (2006); Byrd et al. (2006); Gable et al. (2008); Petter et al. (2008); Hwang and Xu (2008); Yen et al. (2008); McGill and Klobas (2008); Wang and Liao (2008); Al-Adaileh (2008); Lee et al. (2009); Rezaei et al. (2009a, b); Almarashdeh et al. (2010); Klobas and McGill (2010); Adeyinka and Mutula (2010); Jang (2010); Lee et al. (2011); Park et al. (2011); Tella (2012); Chevers et al. (2012); Chen and Cao (2012); Khayun et al. (2012); Leung et al. (2012); Sørum et al. (2012); Kim et al. (2012); Eom et al. (2012); Al-Debei et al. (2013); Balaban et al. (2013); Keramati and Salehi (2013); Hollmann et al. (2013); Petter et al. (2013); Choi et al. (2013); Lwoga (2013); Wang et al. (2014); Mtebe and Raisamo (2014); Ghobakhloo and Tang (2015); Cho et al. (2015); Xinli (2015); Rouibah et al. (2015); Huang et al. (2015); Sappri et al. (2016); Sebetci and Çetin (2016); Son et al. (2016); Borena and Negash (2016); Chiu et al. (2016); Jagannathan et al. (2016); Wang et al. (2016); Ojo (2017); Gaardboe et al. (2017); Assegaff et al. (2017); van Cauter et al. (2017); Shagari et al. (2017); Ramírez-Correa et al. (2017); Abrego Almazán et al. (2017); Lin et al. (2017); Laumer et al. (2017); Suh et al. (2017); Duan et al. (2017); Ji-fan Ren et al. (2017); Nugroho and Prasetyo (2018); Yakubu and Dasuki (2018); Daghouri et al. (2018); Aminah et al. (2018); Arenas-Gaitán et al. (2018; Goeke et al. (2018); Aldholay et al. (2018a, b); Chatterjee et al. (2018); Widjaja et al. (2018); Efiloğlu Kurt (2019); Aldholay et al. (2019); Martins et al. (2019); Wang et al. (2019); Al-Mamary (2019); Doleck et al. (2019); Harr et al. (2019); Tam et al. (2019); Barreiras et al. (2019); Motiwalla et al. (2019); Al-Fraihat et al. (2020); Lwoga et al. (2020); Bradford et al. (2020); Khayer et al. (2020); |
| *User Satisfaction* | Gatian (1994); Rainer Jr. and Watson (1995); Li (1997); Seddon (1997); Drury and Farhoomand (1998); Gelderman (1997); Skok et al. (2001); Rai et al. (2002); McGill et al. (2003); DeLone and McLean (2004); Doll et al. (2004); Landrum and Prybutok (2004); Liu et al. (2005); Almutairi and Subramanina (2005); Iivari (2005); Wu and Wang (2006); Lin and Hee (2006); Sabherwal et al. (2006); Kulkarni et al. (2006); Jang et al. (2006); Hussein et al. (2007); Lin (2007); Landrum et al. (2008); Petter et al. (2008); McGill and Klobas (2008); Wang (2008); Wang and Liao (2008); Lee et al. (2009); Chen and Cheng (2009); Floroupolos et al. (2010); Almarashdeh et al. (2010); Landrum et al. (2010); Klobas and McGill (2010); Fleischman et al. (2010); Adeyinka and Mutula (2010); Jang (2010); Lee et al. (2011); Park et al. (2011); Tella (2012); Chevers et al. (2012); Chen and Cao (2012); Yeh and Tao (2012); Khayun et al. (2012); Leung et al. (2012); Sørum et al. (2012); Kim et al. (2012); Eom et al. (2012); Al-Debei et al. (2013); Balaban et al. (2013); Keramati and Salehi (2013); Hollmann et al. (2013); Petter et al. (2013); Lin et al. (2013); Choi et al. (2013); Lwoga (2013); Poelsman et al. (2013); Gorla and Somers (2014); Lai (2014); Wang et al. (2014); Mtebe and Raisamo (2014); Wang and Lu (2014); Ghobakhloo and Tang (2015); Cho et al. (2015); Xinli (2015); Rouibah et al. (2015); Huang et al. (2015); Hew et al. (2016); Sappri et al. (2016); Sebetci and Çetin (2016); Son et al. (2016); Borena and Negash (2016); Chiu et al. (2016); Jagannathan et al. (2016); Wang et al. (2016); Ojo (2017); Gaardboe et al. (2017); Assegaff et al. (2017); van Cauter et al. (2017); Wie and Widjaja (2017); Ramírez-Correa et al. (2017); Abrego Almazán et al. (2017); Lin et al. (2017); Laumer et al. (2017); Suh et al. (2017); Duan et al. (2017); Nugroho and Prasetyo (2018); Daghouri et al. (2018); Aminah et al. (2018); Arenas-Gaitán et al. (2018; Goeke et al. (2018); Aldholay et al. (2018a, b); Chatterjee et al. (2018); Widjaja et al. (2018); Efiloğlu Kurt (2019); Aldholay et al. (2019); Martins et al. (2019); Wang et al. (2019); Alzahrani et al. (2019); Al-Mamary (2019); Doleck et al. (2019); Harr et al. (2019); Tam et al. (2019); Barreiras et al. (2019); Motiwalla et al. (2019); Alkraiji (2020); Al-Fraihat et al. (2020); Lwoga et al. (2020); Bradford et al. (2020); Yoo (2020); Chang et al. (2020); |

## Cross-case causal networks

This section contains the cross-case causal networks of the Process (Figure S1), Correspondence (Figure S2), Interaction (Figure S3), and Expectation (Figure S4) clusters. It is worth stressing that, even though we were able to develop the Process cross-case causal network, we did not find out any causal link or association between its Conceptual Units referring and the ERP benefits flow. Yet, we included it in this Supplementary Material file for anyone who may find it useful for other research purposes.

Each Conceptual Unit in these networks is a cause/effect and is marked with the letter corresponding to the case(s) which such cause/effect was detected in. For example, let us consider Figure S1. "*Definition of trade-offs _AB_*" – a Conceptual Unit emerged from cases A and B – concerns the need to balance the requests from the different business functions within the ERP project, since such requests may be at odds with each other. This Conceptual Unit is one of the causes of "*Cross-functional optimum _AC_*" – a Conceptual Unit stemmed from all the four cases (A, B, C, and D) and that refers to the definition of the best solution, from the perspective of the firm as a whole, that takes into account the most meaningful requests by the functional units.


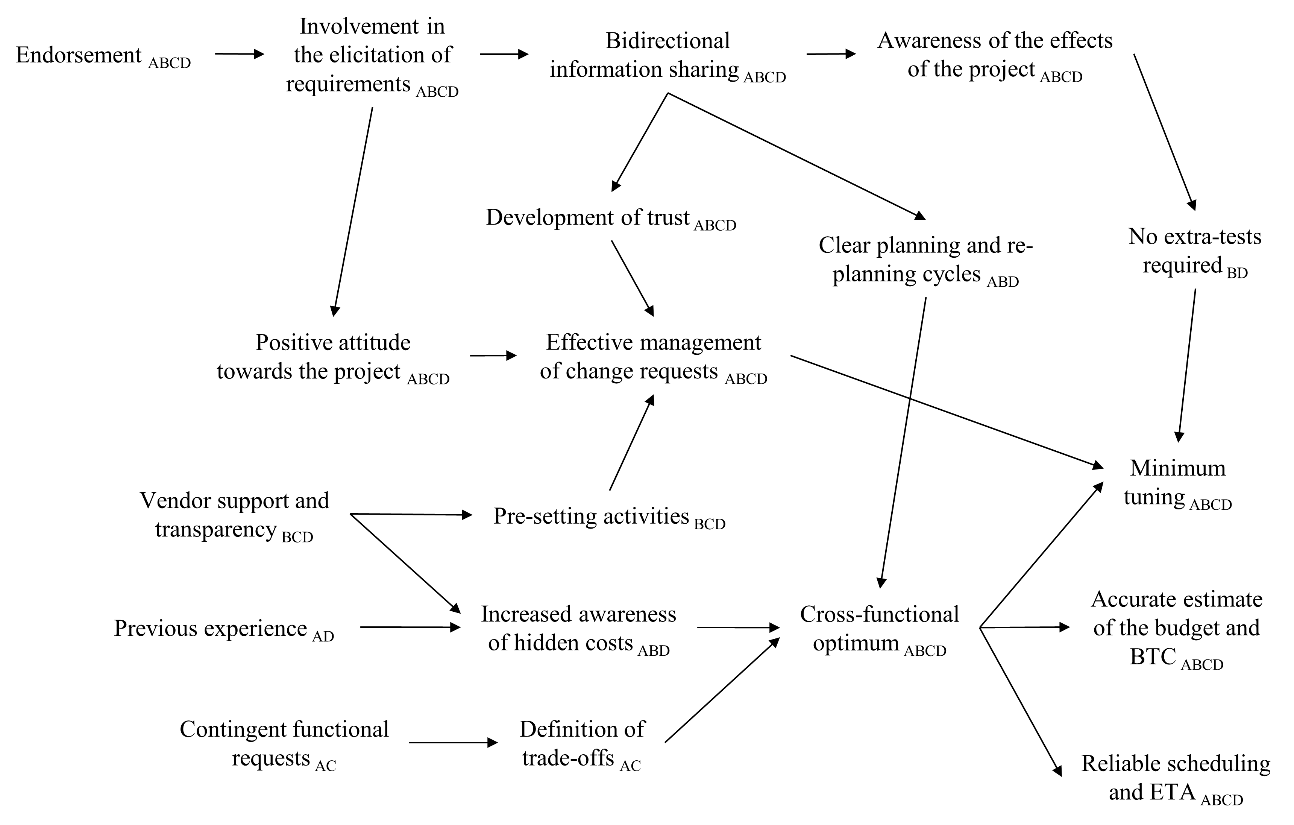


Figure S1. Cross-case causal network of the Process cluster


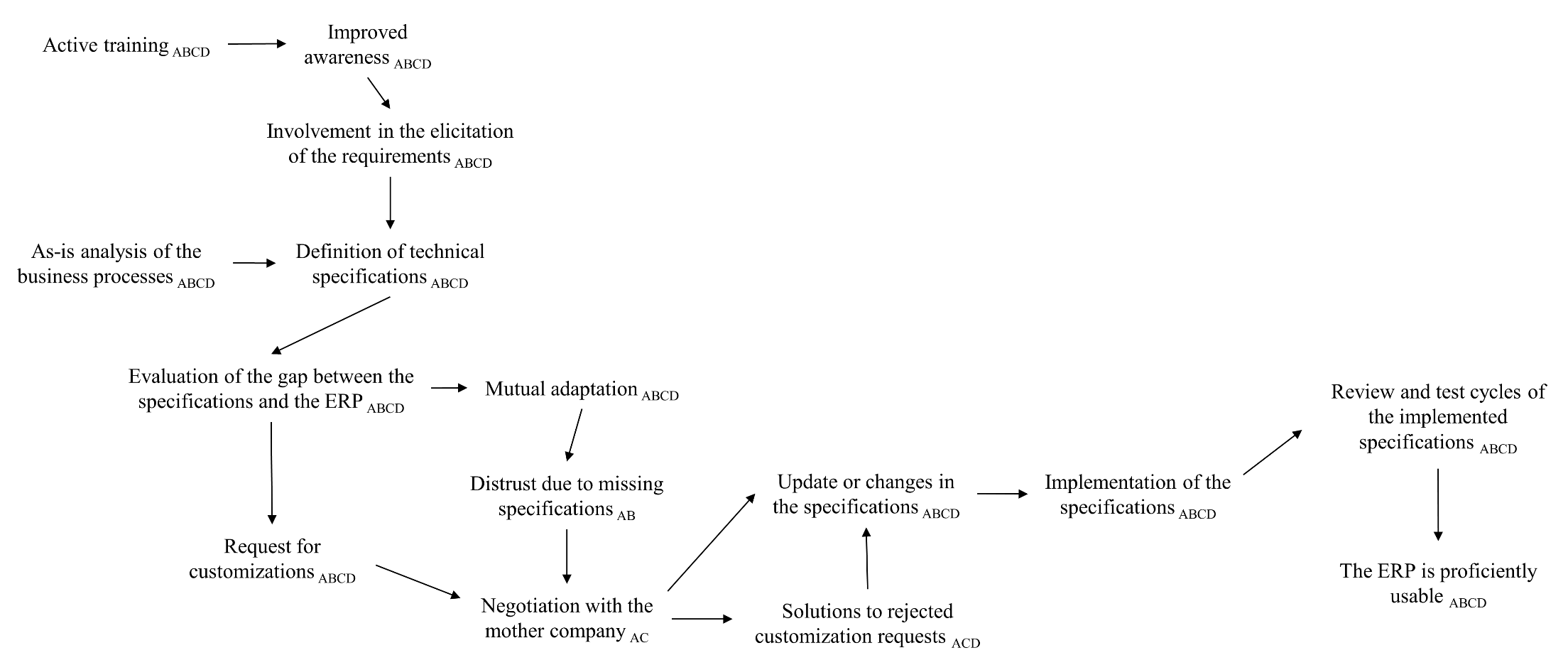


Figure S2. Cross-case causal network of the Correspondence cluster

#
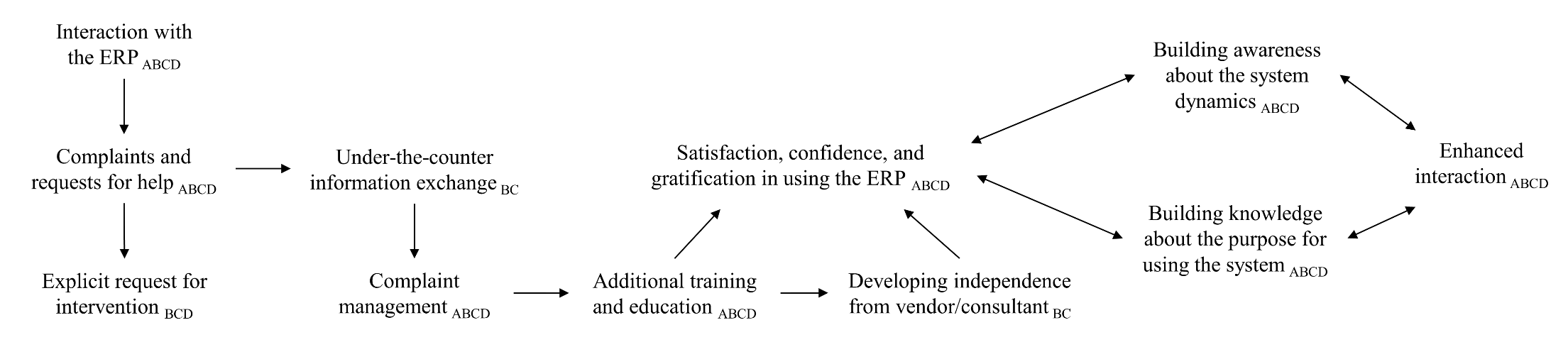


Figure S3. Cross-case causal network of the Interaction cluster


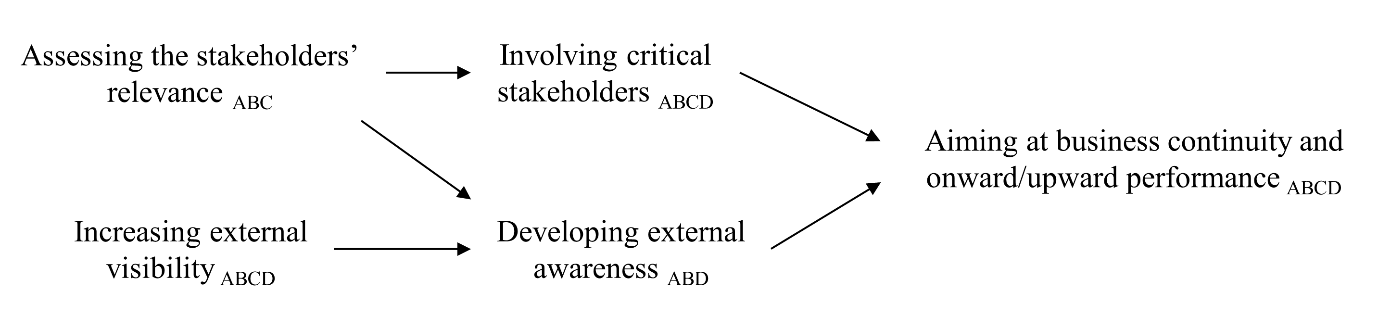


Figure S4. Cross-case causal network of the Expectation cluster

## Questions of the semi-structured interviews

This section contains the question stems of the interviews. Since the interviews were semi-structured, the sequence and the formulation of the questions were not considered as fixed and were re-adapted according to the informant (Adams, 2015). Furthermore, each verbal interchange was expanded through follow-up questions (Adams, 2015).

The questions refined after the pilot test are as follows:

1. Which factors and performances may define the success of your ERP implementation initiative? Why? Are they measurable in an objective way? If yes, how?
2. To which extent each factor and performance does contribute to the achievement of the success? And why?
3. Is the project-shakedown-onward/upward implementation structure relevant to the achievement of the ERP post-implementation success? If yes, why?
4. Does performing over-time and over-budget affect your perception of the ERP success? Why?
5. If the ERP requirements were correctly translated into specifications that have been implemented, may the whole implementation be considered successful? And why?
6. Is the consistency between your company work procedures and the ERP work procedures a proxy for the implementation success? Why?
7. Could it happen that an implementation is not satisfactory despite the specifications being correctly implemented? Which may be the main reasons? And why?
8. Does using the system mean that the implementation was a success? Why?
9. Which internal and external stakeholders may be interested in the ERP implementation success? Why should they be taken into account? To which extent they may affect the implementation during the onward/upward phase? And why?

# References

Abrego Almazán, D., Sánchez Tovar, Y., Medina Quintero, J.M. Influence of information systems on organizational results [Influencia de los sistemas de información en los resultados organizacionales] (2017) Contaduria y Administracion, 62 (2), pp. 321-338.

Adams, W.C. (2015), “Conducting Semi-Structured Interviews”, in Newcomer, K.E., Hatry, H.P. and Wholey, J.S. (Eds.), Handbook of Practical Program Evaluation, 4th Editio., Jossey-Bass.

Adeyinka, T., Mutula, S. A proposed model for evaluating the success of WebCT course content management system (2010) Computers in Human Behavior, 26 (6), pp. 1795-1805.

Al-Adaileh, R.M. An evaluation of information systems success: A user perspective - the case of jordan telecom group (2009) European Journal of Scientific Research, 37 (2), pp. 226-239.

Al-Debei, M.M., Jalal, D., Al-Lozi, E. Measuring web portals success: A respecification and validation of the DeLone and McLean information systems success model (2013) International Journal of Business Information Systems, 14 (1), pp. 96-133.

Aldholay, A., Abdullah, Z., Isaac, O., Mutahar, A.M. Perspective of Yemeni students on use of online learning: Extending the information systems success model with transformational leadership and compatibility (2019) Information Technology and People, 33 (1), pp. 106-128.

Aldholay, A., Isaac, O., Abdullah, Z., Abdulsalam, R., Al-Shibami, A.H. An extension of Delone and McLean IS success model with self-efficacy: Online learning usage in Yemen (2018b) International Journal of Information and Learning Technology, 35 (4), pp. 285-304.

Aldholay, A.H., Isaac, O., Abdullah, Z., Ramayah, T. The role of transformational leadership as a mediating variable in DeLone and McLean information system success model: The context of online learning usage in Yemen (2018a) Telematics and Informatics, 35 (5), pp. 1421-1437.

Al-Fraihat, D., Joy, M., Masa'deh, R., Sinclair, J. Evaluating E-learning systems success: An empirical study (2020) Computers in Human Behavior, 102, pp. 67-86.

Alkraiji, A.I. An examination of citizen satisfaction with mandatory e-government services: comparison of two information systems success models (2020) Transforming Government: People, Process and Policy.

Al-Mamary, Y.H.S. Measuring information systems success in yemen: Potential of delone and mcleans model (2019) International Journal of Scientific and Technology Research, 8 (7), pp. 793-799.

Almarashdeh, I.A., Sahari, N., Zin, N.A.M., Alsmadi, M. The success of learning management system among distance learners in Malaysian universities (2010) Journal of Theoretical and Applied Information Technology, 21 (2), pp. 80-91.

Almutairi, H., Subramanian, G.H. An empirical application of the DeLone and Mclean model in the Kuwaiti private sector (2005) Journal of Computer Information Systems, 45 (3), pp. 113-122.

Alzahrani, A.I., Mahmud, I., Ramayah, T., Alfarraj, O., Alalwan, N. Modelling digital library success using the DeLone and McLean information system success model (2019) Journal of Librarianship and Information Science, 51 (2), pp. 291-306.

Aminah, S., Ditari, Y., Kumaralalita, L., Hidayanto, A.N., Phusavat, K., Anussornnitisarn, P. E-procurement system success factors and their impact on transparency perceptions: Perspectives from the supplier side (2018) Electronic Government, 14 (2), pp. 177-199.

Arenas-Gaitán, J., Rondán-Cataluña, F.J., Ramírez-Correa, P.E. Modelling the success of learning management systems: application of latent class segmentation using FIMIX-PLS (2018) Interactive Learning Environments, 26 (1), pp. 135-147.

Assegaff, S., Hendri, Sunoto, A., Yani, H., Kisbiyanti, D. Social media success model for knowledge sharing (Scale development and validation) (2017) Telkomnika (Telecommunication Computing Electronics and Control), 15 (3), pp. 1335-1343.

Ayyash, M.M., Ahmad, K., Singh, D. A hybrid information system model for trust in e-government initiative adoption in public sector organization (2012) International Journal of Business Information Systems, 11 (2), pp. 162-179.

Balaban, I., Mu, E., Divjak, B. Development of an electronic Portfolio system success model: An information systems approach (2013) Computers and Education, 60 (1), pp. 396-411.

Barreiras, N., Correia, F.N., Matos, R.S. Assessment of a Web-Based Water Information System Performance in the Context of Groundwater Governance (2019) Water Resources Management, 33 (14), pp. 4939-4953.

Borena, B., Negash, S. IT Infrastructure Role in the Success of a Banking System: The Case of Limited Broadband Access (2016) Information Technology for Development, 22 (2), pp. 265-278.

Bradford, M., Henderson, D., Baxter, R.J., Navarro, P. Using generalized audit software to detect material misstatements, control deficiencies and fraud: How financial and IT auditors perceive net audit benefits (2020) Managerial Auditing Journal, 35 (4), pp. 521-547.

Bradley, R.V., Pridmore, J.L., Byrd, T.A. Information systems success in the context of different corporate cultural types: An empirical investigation (2006) Journal of Management Information Systems, 23 (2), pp. 267-294.

Byrd, T.A., Thrasher, E.H., Lang, T., Davidson, N.W. A process-oriented perspective of IS success: Examining the impact of IS on operational cost (2006) Omega, 34 (5), pp. 448-460.

Chang, C.-M., Hsu, M.-H., Huang, E., Yang, Y.-H. Measuring social media brand community success: the roles of media capability and organisational support (2020) Total Quality Management and Business Excellence, 31 (13-14), pp. 1454-1466.

Chatterjee, S., Kar, A.K., Gupta, M.P. Success of IoT in Smart Cities of India: An empirical analysis (2018) Government Information Quarterly, 35 (3), pp. 349-361.

Chen, C.-W.D., Cheng, C.-Y.J. Understanding consumer intention in online shopping: A respecification and validation of the DeLone and McLean model (2009) Behaviour and Information Technology, 28 (4), pp. 335-345.

Chen, H.-J., Kao, C.-H. Empirical validation of the importance of employees' learning motivation for workplace e-learning in Taiwanese organisations (2012) Australasian Journal of Educational Technology, 28 (4), pp. 580-598.

Chevers, D.A., Duggan, E.W., Moore, S. The contribution of process, people and perception to information systems quality and success: A jamaican study (2012) Electronic Journal of Information Systems in Developing Countries, 55 (1), art. no. 4, .

Chiu, P.-S., Chao, I.-C., Kao, C.-C., Pu, Y.-H., Huang, Y.-M. Implementation and evaluation of mobile e-books in a cloud bookcase using the information system success model (2016) Library Hi Tech, 34 (2), pp. 207-223.

Cho, K.W., Bae, S.-K., Ryu, J.-H., Kim, K.N., An, C.-H., Chae, Y.M. Performance evaluation of public hospital information systems by the information system success model (2015) Healthcare Informatics Research, 21 (1), pp. 43-48.

Choi, W., Rho, M.J., Park, J., Kim, K.-J., Kwon, Y.D., Choi, I.Y. Information system success model for customer relationship management system in health promotion centers (2013) Healthcare Informatics Research, 19 (2), pp. 110-120.

Chung, N., Kwon, S.J. Effect of trust level on mobile banking satisfaction: A multi-group analysis of information system success instruments (2009) Behaviour and Information Technology, 28 (6), pp. 549-562.

Chung, N., Lee, H., Lee, S.J., Koo, C. The influence of tourism website on tourists' behavior to determine destination selection: A case study of creative economy in Korea (2015) Technological Forecasting and Social Change, 96, pp. 130-143.

Crowston, K., Howison, J., Annabi, H. Information systems success in free and open source software development: Theory and measures (2006) Software Process Improvement and Practice, 11 (2), pp. 123-148.

Daghouri, A., Mansouri, K., Qbadou, M. Information system evaluation based on multi-criteria decision making: A comparison of two sectors (2018) International Journal of Advanced Computer Science and Applications, 9 (6), pp. 291-297.

DeLone, W.H., McLean, E.R. Measuring e-commerce success: Applying the DeLone and McLean Information Systems Success Model (2004) International Journal of Electronic Commerce, 9 (1), pp. 31-47.

Doleck, T., Lajoie, S.P., Bazelais, P. Social networking and academic performance: A net benefits perspective (2019) Education and Information Technologies, 24 (5), pp. 3053-3073.

Doll, W.J., Deng, X., Raghunathan, T.S., Torkzadeh, G., Xia, W. The meaning and measurement of user satisfaction: A multigroup invariance analysis of the end-user computing satisfaction instrument (2004) Journal of Management Information Systems, 21 (1), pp. 227-262.

Drury, D.H., Farhoomand, A.F. A hierarchical structural model of information systems success (1998) INFOR, 36 (1-2), pp. 25-40.

Duan, Y., Miao, M., Wang, R., Fu, Z., Xu, M. A framework for the successful implementation of food traceability systems in China (2017) Information Society, 33 (4), pp. 226-242.

Efiloğlu Kurt, Ö. Examining an e-learning system through the lens of the information systems success model: Empirical evidence from Italy (2019) Education and Information Technologies, 24 (2), pp. 1173-1184.

Eom, S., Ashill, N.J., Arbaugh, J.B., Stapleton, J.L. The role of information technology in e-learning systems success (2012) Human Systems Management, 31 (3-4), pp. 147-163.

Fleischman, G., Walker, K., Johnson, E. A field study of user versus provider perceptions of management accounting system services (2010) International Journal of Accounting & Information Management, 18 (3), pp. 252-285.

Floropoulos, J., Spathis, C., Halvatzis, D., Tsipouridou, M. Measuring the success of the Greek Taxation Information System (2010) International Journal of Information Management, 30 (1), pp. 47-56.

Gaardboe, R., Sandalgaard, N., Nyvang, T. An assessment of business intelligence in public hospitals (2017) International Journal of Information Systems and Project Management, 5 (4), pp. 5-18.

Gable, G.G., Sedera, D., Chan, T. Re-conceptualizing information system success: The IS-impact measurement model (2008) Journal of the Association for Information Systems, 9 (7), pp. 377-408.

Gatian, A.W., Is user satisfaction a valid measure of system effectiveness? (1994) Information and Management, 26 (3), pp. 119-131.

Gelderman, M. The relation between user satisfaction, usage of information systems and performance (1998) Information and Management, 34 (1), pp. 11-18.

Ghobakhloo, M., Tang, S.H. Information system success among manufacturing SMEs: case of developing countries (2015) Information Technology for Development, 21 (4), pp. 573-600.

Goeke, R.J., Crowne, K.A., Laker, D.R. The Effect of Education on Information Systems Success: Lessons from Human Resources (2018) Information Resources Management Journal, 31 (3), pp. 1-16.

Gorla, N., Somers, T.M. The impact of IT outsourcing on information systems success (2014) Information and Management, 51 (3), pp. 320-335.

Harr, A., vom Brocke, J., Urbach, N. Evaluating the individual and organizational impact of enterprise content management systems (2019) Business Process Management Journal, 25 (7), pp. 1413-1440.

Hazen, B.T., Huscroft, J., Hall, D.J., Weigel, F.K., Hanna, J.B. Reverse logistics information system success and the effect of motivation (2014) International Journal of Physical Distribution and Logistics Management, 44 (3), pp. 201-220.

Hew, J.-J., Lee, V.-H., Leong, L.-Y., Hew, T.-S., Ooi, K.-B. The dawning of mobile tourism: What contributes to its system success? (2016) International Journal of Mobile Communications, 14 (2), pp. 170-201.

Hollmann, V., Lee, H., Zo, H., Ciganek, A.P. Examining success factors of open source software repositories: The case of OSOR.eu portal (2013) International Journal of Business Information Systems, 14 (1), pp. 1-20.

Huang, Y.-M., Pu, Y.-H., Chen, T.-S., Chiu, P.-S. Development and evaluation of the mobile library service system success model A case study of Taiwan (2015) Electronic Library, 33 (6), pp. 1174-1192.

Hussein, R., Karim, N.S.A., Hasan Selamat, M. The impact of technological factors on information systems success in the electronic-government context (2007) Business Process Management Journal, 13 (5), pp. 613-627.

Hwang, M.I., Xu, H. A structural model of data warehousing success (2008) Journal of Computer Information Systems, 49 (1), pp. 48-56.

Iivari, J. An Empirical Test of the DeLone-McLean Model of Information System Success (2005) Data Base for Advances in Information Systems, 36 (2), pp. 8-27.

Ishman, M.D., Carl Pegels, C., Lawrence Sanders, G. Managerial information system success factors within the cultural context of North America and a former Soviet Republic (2001) Journal of Strategic Information Systems, 10 (4), pp. 291-312.

Jagannathan, V., Balasubramanian, S., Natarajan, T. A modified approach for Information Systems Success in the context of Internet Banking using structural equation modelling with R: An empirical study from India (2016) International Journal of e-Business Research, 12 (3), pp. 26-43.

Jang, C.-L. Measuring electronic government procurement success and testing for the moderating effect of computer self-efficacy (2010) International Journal of Digital Content Technology and its Applications, 4 (3).

Jang, J.-H., Kim, J.-K., Hwang, Y.-H. Influence of hotel information system quality on system use and user satisfaction (2006) Journal of Quality Assurance in Hospitality and Tourism, 7 (3), pp. 41-58.

Ji-fan Ren, S., Fosso Wamba, S., Akter, S., Dubey, R., Childe, S.J. Modelling quality dynamics, business value and firm performance in a big data analytics environment (2017) International Journal of Production Research, 55 (17), pp. 5011-5026.

Keramati, A., Salehi, M. Website success comparison in the context of e-recruitment: An analytic network process (ANP) approach (2013) Applied Soft Computing Journal, 13 (1), pp. 173-180.

Khayer, A., Bao, Y., Nguyen, B. Understanding cloud computing success and its impact on firm performance: an integrated approach (2020) Industrial Management and Data Systems, 120 (5), pp. 963-985.

Khayun, V., Ractham, P., Firpo, D. Assessing e-Excise sucess with Delone and Mclean's model (2012) Journal of Computer Information Systems, 52 (3), pp. 31-40.

Kim, C.S., Peterson, D., Kim, J.H. Information systems success: Perceptions of developers in Korea (1999a) Journal of Computer Information Systems, 40 (2), pp. 90-95.

Kim, C.S., Peterson, D., Meinert, D. Student's perceptions on information systems success (1999b) Journal of Computer Information Systems, 39 (3), pp. 68-72.

Kim, C.S., Peterson, D.K. Cultural differences in developers' perceptions of information systems success factors: Japan vs. the United States (2002) Journal of Global Information Management, 10 (2), pp. 5-13.

Kim, K., Trimi, S., Park, H., Rhee, S. The Impact of CMS Quality on the Outcomes of E-learning Systems in Higher Education: An Empirical Study (2012) Decision Sciences Journal of Innovative Education, 10 (4), pp. 575-587.

Klobas, J.E., McGill, T.J. The role of involvement in learning management system success (2010) Journal of Computing in Higher Education, 22 (2), pp. 114-134.

Kulkarni, U.R., Ravindran, S., Freeze, R. A knowledge management success model: Theoretical development and empirical validation (2006) Journal of Management Information Systems, 23 (3), pp. 309-347.

Lai, J.-Y. E-SERVCON and e-commerce success: Applying the DeLone & McLean model (2014) Journal of Organizational and End User Computing, 26 (3), pp. 1-22.

Landrum, H., Prybutok, V.R. A service quality and success model for the information service industry (2004) European Journal of Operational Research, 156 (3), pp. 628-642.

Landrum, H., Prybutok, V.R., Zhang, X. The moderating effect of occupation on the perception of information services quality and success (2010) Computers and Industrial Engineering, 58 (1), pp. 133-142.

Landrum, H.T., Prybutok, V.R., Strutton, D., Zhang, X. Examining the merits of usefulness versus use in an information service quality and information system success web-based model (2008) Information Resources Management Journal, 21 (2), pp. 1-17.

Laumer, S., Maier, C., Weitzel, T. Information quality, user satisfaction, and the manifestation of workarounds: A qualitative and quantitative study of enterprise content management system users (2017) European Journal of Information Systems, 26 (4), pp. 333-360.

Lee, J., Bharosa, N., Yang, J., Janssen, M., Rao, H.R. Group value and intention to use - A study of multi-agency disaster management information systems for public safety (2011) Decision Support Systems, 50 (2), pp. 404-414.

Lee, S.-Y.T., Kim, H.-W., Gupta, S. Measuring open source software success (2009) Omega, 37 (2), pp. 426-438.

Leung, Z.C.S., Cheung, C.F., Chan, K.T., Lo, K.H.K. Effectiveness of knowledge management systems in social services: Food assistance project as an example (2012) Administration in Social Work, 36 (3), pp. 302-313.

Li, E.Y. Perceived importance of information system success factors: A meta analysis of group differences (1997) Information and Management, 32 (1), pp. 15-28.

Lin, H.-C., Yang, C.-W., Chiou, J.-Y. Physicians' participation in practice of knowledge management systems (2013) Journal of Information and Knowledge Management, 12 (2), art. no. 1350012, .

Lin, H.-F. Measuring online learning systems success: Applying the updated DeLone and McLean model (2007) Cyberpsychology and Behavior, 10 (6), pp. 817-820.

Lin, H.-F., Lee, G.-G. Determinants of success for online communities: An empirical study (2006) Behaviour and Information Technology, 25 (6), pp. 479-488.

Lin, H.-H., Wang, Y.-S., Li, C.-R., Shih, Y.-W., Lin, S.-J. The Measurement and Dimensionality of Mobile Learning Systems Success (2017) Journal of Educational Computing Research, 55 (4), pp. 449-470.

Lin, W.-S., Wang, C.-H. Antecedences to continued intentions of adopting e-learning system in blended learning instruction: A contingency framework based on models of information system success and task-technology fit (2012) Computers and Education, 58 (1), pp. 88-99.

Liu, S.-C., Olfman, L., Ryan, T. Knowledge Management System Success: Empirical Assessment of a Theoretical Model (2005) International Journal of Knowledge Management (IJKM), 1 (2), pp. 68-87.

Lwoga, E.T. Measuring the success of library 2.0 technologies in the African context: The suitability of the DeLone and McLean's modelm (2013) Campus-Wide Information Systems, 30 (4), pp. 288-307.

Lwoga, E.T., Sangeda, R.Z., Mushi, R. Predictors of electronic health management information system for improving the quality of care for women and people with disabilities (2020) Information Development.

Martins, J., Branco, F., Gonçalves, R., Au-Yong-Oliveira, M., Oliveira, T., Naranjo-Zolotov, M., Cruz-Jesus, F. Assessing the success behind the use of education management information systems in higher education (2019) Telematics and Informatics, 38, pp. 182-193.

Martinsons, M.G., Chong, P.K.C. The influence of human factors and specialist involvement on information systems success (1999) Human Relations, 52 (1), pp. 123-152.

McGill, T., Hobbs, V., Klobas, J. User-developed applications and information systems success: A test of DeLone and McLean's model (2003) Information Resources Management Journal, 16 (1), pp. 24-45.

McGill, T., Klobas, J. User developed application success: Sources and effects of involvement (2008) Behaviour and Information Technology, 27 (5), pp. 407-422.

Motiwalla, L.F., Albashrawi, M., Kartal, H.B. Uncovering unobserved heterogeneity bias: Measuring mobile banking system success (2019) International Journal of Information Management, 49, pp. 439-451.

Mtebe, J.S., Raisamo, R. A model for assessing learning management system success in higher education in sub-saharan Countries (2014) Electronic Journal of Information Systems in Developing Countries, 61 (1), art. no. 7, .

Nugroho, Y., Prasetyo, A. Assessing information systems success: A respecification of the DeLone and McLean model to integrating the perceived quality (2018) Problems and Perspectives in Management, 16 (1), pp. 348-360.

Ojo, A.I. Validation of the delone and mclean information systems success model (2017) Healthcare Informatics Research, 23 (1), pp. 60-66.

Ong, C.-S., Day, M.-Y., Hsu, W.-L. The measurement of user satisfaction with question answering systems (2009) Information and Management, 46 (7), pp. 397-403.

Park, S., Zo, H., Ciganek, A.P., Lim, G.G. Examining success factors in the adoption of digital object identifier systems (2011) Electronic Commerce Research and Applications, 10 (6), pp. 626-636.

Petter, S., DeLone, W., McLean, E. Measuring information systems success: Models, dimensions, measures, and interrelationships (2008) European Journal of Information Systems, 17 (3), pp. 236-263.

Petter, S., Delone, W., McLean, E.R. Information systems success: The quest for the independent variables (2013) Journal of Management Information Systems, 29 (4), pp. 7-62.

Poelmans, S., Reijers, H.A., Recker, J. Investigating the success of operational business process management systems (2013) Information Technology and Management, 14 (4), pp. 295-314.

Popovič, A., Hackney, R., Coelho, P.S., Jaklič, J. How information-sharing values influence the use of information systems: An investigation in the business Intelligence systems context (2014) Journal of Strategic Information Systems, 23 (4), pp. 270-283.

Rai, A., Lang, S.S., Welker, R.B. Assessing the validity of IS success models: An empirical test and theoretical analysis (2002) Information Systems Research, 13 (1), pp. 50-69.

Rainer Jr., R.K., Watson, H.J., The keys to executive information system success (1995) Journal of Management Information Systems, 12 (2), pp. 83-98.

Ramírez-Correa, P.E., Rondan-Cataluña, F.J., Arenas-Gaitán, J., Alfaro-Perez, J.L. Moderating effect of learning styles on a learning management system's success (2017) Telematics and Informatics, 34 (1), pp. 272-286.

Rezaei, A., Asadi, A., Rezvanfar, A., Hassanshahi, H. The impact of organizational factors on management information system success: An investigation in the Iran's agricultural extension providers (2009) International Information and Library Review, 41 (3), pp. 163-172.

Rezaei, A., Asadi, A., Rezvanfar, A., Hassanshahi, H. The impact of organizational factors on management information system success: An investigation in the Iran's agricultural extension providers (2009) International Information and Library Review, 41 (3), pp. 163-172.

Rouibah, K., Lowry, P.B., Almutairi, L. Dimensions of business-to-consumer (B2C) systems success in Kuwait: Testing a modified DeLone and McLean IS success model in an e-commerce context (2015) Journal of Global Information Management, 23 (3), pp. 41-71.

Saarinen, T. An expanded instrument for evaluating information system success (1996) Information and Management, 31 (2), pp. 103-118.

Sabherwal, R., Jeyaraj, A., Chowa, C. Information system success: Individual and organizational determinants (2006) Management Science, 52 (12), pp. 1849-1864.

Sappri, M.M., Baharudin, A.S., Raman, S. The moderating effect of user involvement and self-readiness and factors that influence information system net benefits among malaysian public sector (2016) International Journal of Applied Engineering Research, 11 (18), pp. 9659-9673.

Sebetci, O., Çetin, M. Developing, applying and measuring an e-Prescription Information Systems Success Model from the perspectives of physicians and pharmacists (2016) Health Policy and Technology, 5 (1), pp. 84-93.

Seddon, P.B. A Respecification and Extension of the DeLone and McLean Model of IS Success (1997) Information Systems Research, 8 (3), pp. 240-253.

Shagari, S.L., Abdullah, A., Saat, R.M. Accounting information systems effectiveness: Evidence from the Nigerian banking sector (2017) Interdisciplinary Journal of Information, Knowledge, and Management, 12, pp. 309-335.

Skok, W., Kophamel, A., Richardson, I. Diagnosing information systems success: Importance-performance maps in the health club industry (2001) Information and Management, 38 (7), pp. 409-419.

Son, H., Hwang, N., Kim, C., Cho, Y. Construction professionals’ perceived benefits of PMIS: The effects of PMIS quality and computer self-efficacy (2016) KSCE Journal of Civil Engineering, 20 (2), pp. 564-570.

Sørum, H., Medaglia, R., Andersen, K.N., Scott, M., DeLone, W. Perceptions of information system success in the public sector: Webmasters at the steering wheel? (2012) Transforming Government: People, Process and Policy, 6 (3), pp. 239-257.

Suh, H., Chung, S., Choi, J. An empirical analysis of a maturity model to assess information system success: a firm-level perspective (2017) Behaviour and Information Technology, 36 (8), pp. 792-808.

Szajna, B., Determining information system usage: Some issues and examples (1993) Information and Management, 25 (3), pp. 147-154.

Tam, C., Loureiro, A., Oliveira, T. The individual performance outcome behind e-commerce: Integrating information systems success and overall trust (2019) Internet Research, 30 (2), pp. 439-462.

Tella, A. Determinants of E-Payment Systems Success: A User's Satisfaction Perspective (2012) International Journal of E-Adoption, 4 (3), pp. 15-38.

Van Cauter, L., Verlet, D., Snoeck, M., Crompvoets, J. The explanatory power of the Delone & McLean model in the public sector: A mixed method test (2017) Information Polity, 22 (1), pp. 41-55.

Wang, W.-T., Lu, C.-C. Determinants of Success for Online Insurance Web Sites: The Contributions from System Characteristics, Product Complexity, and Trust (2014) Journal of Organizational Computing and Electronic Commerce, 24 (1), pp. 1-35.

Wang, Y.-S. Assessing e-commerce systems success: A respecification and validation of the DeLone and McLean model of IS success (2008) Information Systems Journal, 18 (5), pp. 529-557.

Wang, Y.-S., Li, C.-R., Yeh, C.-H., Cheng, S.-T., Chiou, C.-C., Tang, Y.-C., Tang, T.-I. A conceptual model for assessing blog-based learning system success in the context of business education (2016) International Journal of Management Education, 14 (3), pp. 379-387.

Wang, Y.-S., Li, H.-T., Li, C.-R., Wang, C.A model for assessing blog-based learning systems success (2014) Online Information Review, 38 (7), pp. 969-990.

Wang, Y.-S., Liao, Y.-W. Assessing eGovernment systems success: A validation of the DeLone and McLean model of information systems success (2008) Government Information Quarterly, 25 (4), pp. 717-733.

Wang, Y.-Y., Wang, Y.-S., Lin, H.-H., Tsai, T.-H. Developing and validating a model for assessing paid mobile learning app success (2019) Interactive Learning Environments, 27 (4), pp. 458-477.

Widjaja, A.E., Chen, J.V., Gonchig, B. Investigating factors affecting central bank information systems success: The case of the central bank of Mongolia (2018) International Journal of Technology and Human Interaction, 14 (4), pp. 43-62.

Wie, A.Y.P.L., Widjaja, A.W. B2C E-commerce site success factors: A comparison between Indonesia, Japan, and South Korea (2017) International Journal of Economics and Management, 11 (2 Special Issue), pp. 505-527.

Wu, J.-H., Wang, Y.-M. Measuring KMS success: A respecification of the DeLone and McLean's model (2006) Information and Management, 43 (6), pp. 728-739.

Xinli, H. Effectiveness of information technology in reducing corruption in China (2015) Electronic Library, 33 (1), pp. 52-64.

Yakubu, M.N., Dasuki, S.I. Assessing eLearning systems success In Nigeria: An application of the Delone And Mclean information systems success model (2018) Journal of Information Technology Education: Research, 17, pp. 183-203.

Yeh, C.-C.R., Tao, Y.-H. College students' intention to continue using a personal response system: Deriving a model from four theoretical perspectives (2012) Australasian Journal of Educational Technology, 28 (5), pp. 912-930.

Yen, H.R., Li, E.Y., Niehoff, B.P. Do organizational citizenship behaviors lead to information system success?. Testing the mediation effects of integration climate and project management (2008) Information and Management, 45 (6), pp. 394-402.

Yoo, J. The effects of perceived quality of augmented reality in mobile commerce-an application of the information systems success model (2020) Informatics, 7 (2), art. no. 14.

Zhou, T. Examining the critical success factors of mobile website adoption (2011) Online Information Review, 35 (4), pp. 636-652.
